# DLST Succinylation‐Mediated Mitochondrial Metabolic Remodeling and Cuproptosis Resistance Promote Malignant Progression of Lung Adenocarcinoma

**DOI:** 10.1002/advs.77528

**Published:** 2026-08-30

**Authors:** Xuanxuan Li, Peijun Zhou, Xingzhi Peng, Qixin Liu, Wenhui Zeng, Rui Wang, Liangfang Shen, Jinwu Peng, Qin Zhou, Lifang Yang

**Affiliations:** ^1^ Department of Oncology National Clinical Research Center for Geriatric Disorders, Key Laboratory of Carcinogenesis and Cancer Invasion of Ministry of Education, Xiangya Hospital Central South University Changsha China; ^2^ Cancer Research Institute Xiangya School of Basic Medicine Science Central South University Changsha China; ^3^ Department of Pathology Xiangya Hospital Central South University Changsha China; ^4^ Department of Pathology Xiangya Changde Hospital Changde China

**Keywords:** cuproptosis, DLST, LUAD, metabolic remodeling, succinylation

## Abstract

Metabolic remodeling is one of the hallmarks of malignant tumors, and post‐translational modifications of proteins, such as succinylation, play an important role in the process of metabolic remodeling by regulating the function of metabolic enzymes. However, the role of succinylation modifications of key mitochondrial metabolic enzymes in lung adenocarcinoma (LUAD) remains unclear. Here, using succinylation proteomics, it is demonstrated that the succinylation level of lysine 409 (K409) on dihydrolipoamide S‐succinyltransferase (DLST) is significantly increased in LUAD. Further, DLST K409 succinylation (K409succ), which is catalyzed by carnitine palmitoyltransferase 1A (CPT1A), can affect oxidative phosphorylation (OXPHOS) and redox homeostasis by regulating enzyme activity, thereby promoting the malignant progression of LUAD. Notably, the succinylation of DLST can enhance the cuproptosis resistance by inhibiting its lipoylation. Subsequently, a small‐molecule inhibitor SI409‐1 that can specifically target DLST K409 has been developed. In vitro and in vivo experiments show that SI409‐1 can inhibit the LUAD growth and enhance the sensitivity to cuproptosis inducers. Collectively, these findings provide a novel predictive target and intervention strategy for the clinical diagnosis and treatment of LUAD.

## Graphical abstract

The DLST K409 succinylation mediated by CPT1A promotes OXPHOS and redox homeostasis through regulating enzyme activity, thereby promoting the malignant progression of LUAD. Notably, the succinylation of DLST can enhance the cuproptosis resistance by inhibiting its lipoylation. Further, small‐molecule inhibitor SI409‐1 that specifically target the DLST K409 can inhibit the progression of LUAD.

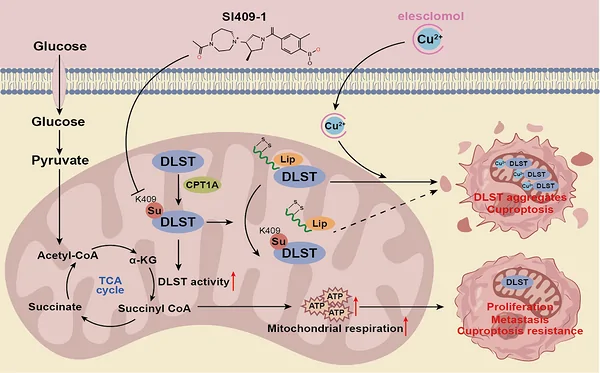

## 1 Introduction

Lung cancer is the most prevalent malignant tumor globally, accounting for 12.4% of all tumor cases and representing 18.7% of tumor‐related deaths worldwide [1]. Non‐small cell lung cancer (NSCLC) accounts for roughly 85% of all lung cancer cases [2]. Among these, lung adenocarcinoma (LUAD), the most prevalent histological subtype of NSCLC, represents 55–60% of all NSCLC patients [3]. Although recent clinical advancements in LUAD treatment, most patients are still diagnosed at an advanced stage (stage III/IV) initially, with approximately 57% of patients having metastatic disease at the time of diagnosis, and the average 5‐year survival rate is merely 6% [4, 5]. Therefore, studying the molecular mechanisms underlying LUAD progression is critical for improving early diagnosis, developing targeted therapies, and enhancing clinical outcomes.

Metabolic remodeling is a key hallmark of tumor cells. Mitochondria, core players in cellular metabolism, generate ATP, NADPH, and macromolecules (nucleotides, lipids, amino acids) via the tricarboxylic acid (TCA) cycle, electron transport chain (ETC), and oxidative phosphorylation (OXPHOS) to regulate energy production, redox homeostasis, and macromolecular synthesis [6]. The Warburg effect was previously considered the primary metabolic pathway of tumor cells, but some studies have also found that inhibiting glycolysis does not prevent the occurrence of certain tumors. For example, in BRCA1‐deficient breast cancer models, knocking out PKM2, the rate‐limiting enzyme in glycolysis, still promotes tumor development [7]. Another study demonstrated that blocking LDHA enhances mitochondrial respiration in breast cancer cells, confirming the persistence of oxidative metabolism [8]. Growing evidence indicates that specific tumor subtypes primarily rely on mitochondrial pathways rather than glycolysis for malignant progression [9]. In pancreatic ductal adenocarcinoma, dormant tumor cells require strong reliance on mitochondrial respiration and reduced glycolytic activity to survive and induce tumor recurrence. These cells exhibit heightened sensitivity to OXPHOS inhibitors [10]. Exosomes carrying circ_0001610 promote PGC‐1a expression by inhibiting miR‐30e‐5p, inducing metabolic remodeling toward OXPHOS. This leads to enhanced stem cell‐like traits and chemotherapy resistance in colorectal cancer [11]. Han et al.’s study using intraoperative 13^C^‐glucose labeling revealed metabolic differences between tumors and normal lung tissue in NSCLC, demonstrating increased reliance on glucose metabolism and OXPHOS [12]. These findings indicate that mitochondrial metabolic remodeling plays a pivotal role in the malignant progression of tumors including LUAD.

The changes in the expression and activity of metabolic enzymes are key regulatory factors in the metabolic remodeling of tumor cells, which are mainly regulated by metabolites, transcription factors, and epigenetic modifications. Among these, post‐translational modifications (PTMs) are important epigenetic regulatory mechanisms that affect the function of metabolic enzymes [13]. Lysine succinylation is a novel PTM closely associated with mitochondrial metabolism, wherein the acyl donor succinyl‐coenzyme A (succinyl‐CoA) covalently attaches a succinyl group to the lysine residues of substrate proteins through enzymatic (succinyltransferases and desuccinylases) or non‐enzymatic means [14]. Compared with methylation and acetylation, succinylation causes a change of two charges in the lysine group, exerting a more significant impact on protein structure and function [15]. Recent studies have found that succinylation modification plays an important role in the proliferation and metastasis of malignant tumors through metabolic remodeling. For example, succinylation of glutaminase (GLS) at K311 enhances its enzymatic activity, thereby increasing glutamate metabolism, as well as the production of NADPH and glutathione, maintaining redox balance and promoting malignant tumor progression [16]. As a newly identified succinyltransferase, 3‐oxoacid CoA‐transferase 1 (OXCT1) catalyzes the succinylation of serine β‐lactamase‐like protein (LACTB) at K284, which in turn inhibits the proteolytic activity of LACTB, leading to an increase in mitochondrial membrane potential and OXPHOS, and ultimately promoting the malignant proliferation and metastasis of hepatocellular carcinoma [17]. Glutamine deprivation induces SIRT5‐mediated desuccinylation of mitochondrial malic enzyme 2 (ME2) at K346, thereby enhancing its enzymatic activity, promoting mitochondrial respiration, and subsequently contributing to the proliferation of colorectal cancer [18]. Succinylation of succinyl‐CoA synthetase GDP‐forming subunit β (SUCLG2) at K93 inhibits TRIM21‐mediated K63‐linked ubiquitin‐lysosome pathway degradation, enhances the stability and enzymatic activity of SUCLG2, further maintaining mitochondrial function and resulting in the malignant proliferation of LUAD [19]. Therefore, succinylation modification can regulate the functions of metabolic enzymes through mechanisms such as modulating enzymatic activity and protein stability, and then participate in the regulation of tumor proliferation and metastasis through metabolic remodeling.

Dihydrolipoamide S‐succinyltransferase (DLST) is the E2 subunit of the α‐ketoglutarate dehydrogenase complex (KGDHC) in the TCA cycle. It facilitates the conversion of α‐ketoglutarate (α‐KG) to succinyl‐CoA, a metabolite essential for cellular energy production and macromolecular synthesis [20]. Studies have identified DLST as a key rate‐limiting enzyme in the TCA cycle, with its high activity contributing to the initiation and progression of various malignant tumors. For example, elevated DLST activity predicts poor overall survival and recurrence‐free survival in patients with triple‐negative breast cancer (TNBC). Knockdown of DLST impairs the growth and migration of TNBC cells with relatively intact TCA cycle function, whereas it exerts minimal effects on TNBC cells with defective TCA cycle function [21]. High DLST activity enhances cellular OXPHOS levels, thereby promoting the growth and invasion of neuroblastoma cells [22]. These findings suggest that DLST may play a critical role in tumor malignant progression by regulating mitochondrial metabolism. However, the mechanisms underlying the regulation of DLST activity remain elusive.

In this study, we found that the succinylation level of K409 of DLST was significantly higher in LUAD tissues than in adjacent normal tissues, and this modification promoted the malignant progression of LUAD. Desuccinylation of DLST K409 could inhibit its enzymatic activity and suppress mitochondrial metabolism in tumor cells. Further, we identified carnitine palmitoyltransferase 1A (CPT1A) as the succinyltransferase for DLST, which enhances DLST K409 succinylation (K409succ). Meanwhile, the succinylation of DLST could enhance the cuproptosis resistance by inhibiting its lipoylation. In addition, we have screened and obtained a small‐molecule inhibitor targeting DLST K409, which could effectively inhibit K409succ, thereby suppressing the malignant progression of LUAD and enhancing cuproptosis sensitivity.

## 2 Materials and Methods

### 2.1 Clinical Samples

The 5 pairs of primary LUAD tissues and their adjacent normal tissues from patients who had not received any preoperative treatment, as well as the 70 LUAD formalin‐fixed paraffin‐embedded samples (Collected  between October 2023 and February 2025), were collected from Xiangya Hospital, Central South University (Changsha, China), and the clinicopathological information for these patients is summarized in Tables S1 and S2.

### 2.2 4D Quantitative Succinylation Proteomics

The LUAD tissue and corresponding adjacent normal tissue samples were sent to PTM Biolabs (Hangzhou, China) for 4D label‐free lysine succinylation proteomics mass spectrometry analysis. The experiment included protein extraction, enzymatic digestion, modification peptide enrichment, and liquid chromatography‐tandem mass spectrometry. Bioinformatics analysis was then performed to annotate quantifiable targets, including protein annotation, functional classification, and enrichment analysis.

### 2.3 Plasmids and Reagents

pCDH‐3×Flag (#167463) and pcDNA3.1‐Flag (#20011) plasmids were obtained from Addgene. The PGEX‐4T‐1 (VT1253), pcDNA3.1‐CPT1A‐His (G104362), PET‐28A‐ CPT1A‐His (NM_001876), PET‐28A‐KAT2A‐His (NM_021078), pcDNA3.1‐LIPT1‐His (NM_015929) and pcDNA3.1‐LIAS‐His (NM_006859) plasmids were purchased from Youbio (Changsha, China). The pLVX‐shRNA vector (632177) was purchased from Clontech (Mountain View, CA). PCR‐amplified DLST sequence was cloned into pCDH‐3×Flag, pcDNA3.1‐Flag, and PGEX‐4T‐1 vectors using a homologous recombination kit (C112‐02, Vazyme, Nanjing, China) to generate DLST overexpression plasmids (pCDH‐3×Flag‐DLST, pcDNA3.1‐Flag‐DLST, GST‐DLST, respectively). Mutant plasmids pCDH‐3×Flag‐DLST K409R and pCDH‐3×Flag‐DLST K409E were constructed using the ClonExpress II One‐Step Cloning Kit (C215, Vazyme). Subsequently, pcDNA3.1‐Flag‐DLST K409R and pcDNA3.1‐Flag‐DLST K409E were generated using the above K409R/E mutants as templates, while GST‐DLST‐K409R was constructed with GST‐wild‐type (WT) as the template. The His‐CPT1A H473A mutant was amplified using His‐CPT1A as a template with the point mutant kit (C215, Vazyme). Primer sequences for plasmid construction are listed in Table S3. shRNAs targeting DLST and CPT1A were synthesized by Tsingke Biotechnology (Beijing, China) and cloned into the pLVX‐shRNA vector to generate shDLST and shCPT1A plasmids (primer sequences in Table S4). All expression constructs were verified by DNA sequencing.

Dimethylsuccinate (HY‐Y0808), Elesclomol (ES, HY‐12040), Ferrostatin‐1 (HY‐100579), Tetrathiomolybdate (TTM, HY‐W076067), Necrostatin‐1 (HY‐15760), Z‐VAD‐FMK (HY‐16658B), SI409‐1, SI409‐2, and SI409‐3 were purchased from MedChemExpress (MCE, Monmouth Junction, NJ).

### 2.4 Cell Culture and Transfection

H1299, A549, H358, and HEK 293T cells were obtained from the Cell Center of Central South University. H1299, A549, and H358 cells were cultured in Dulbecco's Modified Eagle Medium (DMEM)(C11995500BT, Invitrogen, Carlsbad, CA) supplemented with 10% bovine calf serum (BCS)(B7446, Sigma‐Aldrich, Burlington, MA). Human bronchial epithelial cell line BEAS‐2B were cultured in DMEM supplemented with 10% fetal bovine serum (FBS) (F8687, Sigma‐Aldrich). Penicillin‐streptomycin (C3420‐0100, Viva Cell Biosciences, Shanghai, China) was added to all media at a 1:100 dilution. All cells were incubated in a 37°C incubator with 5% CO_2_. Cells were transfected with the NEOFECT DNA Transfection Reagent (TF20121201, Genomtech, Beijing, China) when reaching 70%–80% confluency.

To establish stable cell lines, 293T cells were co‐transfected with packaging plasmids (Gag‐pol: #14887; pRSV‐Rev: #12253; pCMV‐VSV‐G: #8454, Addgene) and shDLST, shCPT1A, Flag‐DLST‐WT or its mutant plasmids. At 48 h post‐transfection, lentiviral supernatants were collected and used to infect H1299 and A549 cells, with the addition of 8 µg/mL polybrene (BL628A, Biosharp Biotechnology, Shanghai, China) to enhance infection efficiency. Infected cells were then selected using medium containing 2 µg/mL puromycin (ST551, Beyotime, Shanghai, China) for one week to obtain stable cell lines: H1299‐shDLST, H1299‐shDLST/WT, H1299‐shDLST/K409R, H1299‐shDLST/K409E, A549‐shDLST, A549‐shDLST/WT, A549‐shDLST/K409R, A549‐shDLST/K409E, H358‐shDLST, H358‐shDLST/WT, H358‐shDLST/K409R, H1299‐shCPT1A, H1299‐shCPT1A/WT, H1299‐shCPT1A/K409R, H1299‐shCPT1A/K409E, A549‐shCPT1A, A549‐shCPT1A/WT, A549‐shCPT1A/K409R, and A549‐shCPT1A/K409E.

### 2.5 Western Blot and Immunoprecipitation (IP)

Cells were washed with PBS and lysed using IP lysis buffer (P10013, Beyotime) supplemented with 10% protease inhibitor (B14001, MCE). Lysates were centrifuged at 13 000 rpm for 15 min at 4°C, and supernatants were collected as cell lysates. Protein concentrations were determined using a BCA protein assay kit (AR0197, Boster Biotechnology, Wuhan, China) according to the manufacturer's instructions. Samples were mixed with 5× loading buffer and boiled. Proteins were separated by 10% SDS‐PAGE, then transferred to PVDF membranes (IPVH00010, Merck Millipore, Darmstadt, Germany). Membranes were blocked with 5% non‐fat milk for 1 h at room temperature, followed by incubation with primary antibodies overnight at 4°C. After three washes with TBST, membranes were incubated with horseradish peroxidase‐conjugated secondary antibodies for 1 h at room temperature. Protein bands were visualized using an enhanced chemiluminescence detection kit (S6010L, Biosharp).

For the IP experiment, cells in 100 mm culture dishes were lysed on ice for 30 min with 500 µL IP lysis buffer with protease inhibitor. A total of 2 mg protein was mixed with the corresponding antibody and 40 µL Dynabeads Protein G (10004D, Invitrogen), then incubated on a rotating shaker at 4°C for 16 h to facilitate antibody‐target protein binding. After incubation, beads were washed twice with ice‐cold PBS to remove unbound proteins. Finally, beads were resuspended in 40 µL 2× protein loading buffer and boiled to prepare for Western blot analysis.

Primary antibodies used were as follows: Anti‐DLST (#11954) was purchased from CST (Danvers, MA). Anti‐pan‐succinylation (#PTM401) was purchased from PTM Biolabs. The polyclonal rabbit anti‐human DLST K409succ antibody was developed by PTM Biolabs. Anti‐lipoic acid (ab58724) was purchased from Abcam (Cambridge, MA). Anti‐CPT1A (15184‐1‐AP), anti‐HSP70 (10995‐1‐AP), anti‐TOM20 (11802‐1‐AP), anti‐His‐Tag (66005‐1‐IG), anti‐Flag‐tag (20543‐1‐AP), GST‐tag (66001‐2‐Ig) and β‐actin (66009‐1‐Ig) were purchased from Proteintech (Wuhan, China).

### 2.6 In Vitro Succinylation Assay

GST‐vector and GST‐DLST, GST‐DLST‐K409R proteins were expressed in *E. coli* BL21 bacteria strain and purified using the GST Tag Protein Purification Kit (P2262, Beyotime). Bacterially purified GST‐DLST protein and succinyl‐CoA (S1129, Sigma) at various concentrations were added to the in vitro succinylation reaction buffer (50 mM Tris‐HCl, pH 7.4). The mixture was incubated at 37°C for 30 min. Succinylation was detected by Western blot.

To analyze the CPT1A‐dependent succinylation reaction, recombinant CPT1A and KAT2A proteins were purified using a His‐tag protein purification kit (P2226, Beyotime). Purified DLST protein was incubated with active CPT1A or KAT2A in an in vitro succinylation reaction system at 37°C for 30 min. The succinylation level of DLST was then determined by Western blot analysis.

### 2.7 Immunohistochemical (IHC) Analysis

The standard protocol for IHC staining and semi‐quantitative analysis of protein expression was as described previously [23]. Clinical and animal samples underwent IHC staining using a universal two‐step detection kit (PV‐9000, ZSGB‐BIO, Beijing, China) and DAB chromogenic kit (ZLI‐9017, ZSGB‐BIO).

### 2.8 CCK‐8 Assay

Cell viability was assessed with the CCK‐8 kit (C0005, Targetmol, Shanghai, China). Cells from each group were seeded in 96‐well plates and cultured. The detection solution was added per the kit instructions, followed by a 1‐h dark incubation. Absorbance at 450 nm was measured using a microplate reader (BioTek ELx800, Winooski, VT).

### 2.9 Colony Formation Assay

1000–1500 LUAD cells with different treatments were evenly seeded into 6‐well plates and cultured for 14 days. Cell colonies were fixed with 4% polyformaldehyde for 20 min and stained with crystal violet (V5265, Sigma‐Aldrich) for 15 min. Colony numbers were counted, with three replicates per treatment group.

### 2.10 Cell Wound‐Healing Assay

Cells of each group were evenly seeded into 24‐well plates. After the cells adhered to the wall, a sterile 200 µL pipette tip was used to vertically create a scratch on the cell layer at the bottom of each well. Serum‐free medium was then added, and after 24 h of incubation, the wound‐healing process was observed under a microscope.

### 2.11 Transwell Assay

Cells (1 × 10^5^ cells/well) suspended in 200 µL serum‐free medium were seeded into the 8.0 µm upper chamber (353097, Falcon, Corning, NY), which was precoated with Matrigel (354234, Corning). The lower chamber was filled with 10% FBS medium. After 24 h of culture, migrated cells were fixed with 4% paraformaldehyde for 15 min and stained with 0.1% crystal violet (V5265, Sigma‐Aldrich). Cells on the lower membrane were counted in 3 random fields via AMEX‐1200 microscope (AMG, Bothell, WA) and ImageJ was used for quantification.

### 2.12 Xenograft Tumor Experiments

Twenty‐one female athymic nude mice (BALB/c, 5 weeks old) were randomly divided into three groups. A549‐shDLST, A549‐shDLST/WT, and A549‐shDLST/K409R stable cell lines (5 × 10^6^ cells/mouse) suspended in medium containing 10% Matrigel were subcutaneously injected into the mice (*n* = 7 per group). For the ES‐Cu treatment animal model, twenty‐four female athymic nude mice (BALB/c, 5 weeks old) were randomly divided into two groups. Then, H1299‐shDLST/WT and H1299‐shDLST/K409R stable cell lines with 10% Matrigel were subcutaneously injected into mice, respectively. When xenograft tumor volumes reached 80–100 mm^3^, each group of mice was randomly divided into two subgroups and treated with DMSO or ES‐Cu (ES: 5 mg/kg + CuCl_2:_ 0.06 mg/kg), intraperitoneally injection, 4 times a week for 3 consecutive weeks. For the combination therapy animal model, cells with 10% Matrigel were subcutaneously injected into twenty‐four female athymic nude mice (BALB/c, 5 weeks old). When xenograft tumor volumes reached 80–100 mm^3^, mice were randomly divided into four groups and intraperitoneally injected with DMSO, ES‐Cu (ES: 5 mg/kg + CuCl_2:_ 0.06 mg/kg), SI409‐1 (15 mg/kg), or ES‐Cu+ SI409‐1, respectively, once every other day for 3 consecutive weeks [24, 25].

Tumor volumes in the mice were measured every 3 days, calculated using the formula: Volume (mm^3^) = d^2^×D/2, where d and D represent the shortest and longest tumor diameters, respectively. Twenty‐four days after subcutaneous inoculation, mice were euthanized. Tumor tissues were dissected and weighed, then subjected to embedding and IHC staining analysis.

### 2.13 α‐KGDHC Enzyme Activity Assay

The α‐KGDHC activity was determined with the α‐KGDHC Activity Assay Kit (ab185440, Abcam) following the manufacturer's instructions. Briefly, a total of 50 µL of sample lysate was added to each well of a 96‐well plate, positive control wells received control samples (ab185440, Abcam), and blank wells received lysis buffer. Subsequently, 50 µL of working solution was added to each well and mixed thoroughly. The plate was incubated at 37°C, and absorbance at 450 nm was measured using a microplate reader at predetermined time points. α‐KGDHC activity was calculated based on the standard curve.

### 2.14 Content Detection of α‐KG

The determination of α‐KG content was conducted using the α‐Ketoglutarate Content Detection Kit (BC5425, Solarbio, Beijing, China). All experiments were performed according to the manufacturer's instructions, and α‐KG was measured by absorbance at 340 nm using a Microplate Reader (BioTekELx800, Winooski).

### 2.15 Untargeted Metabolite Analysis

1 × 10^6^ cells stably expressing A549‐shDLST/WT or A549‐shDLST/K409R were harvested and sent to Metware Company (Wuhan, China) for untargeted metabolite analysis. Each group contained six parallel samples. For screening differential metabolic substrates, log2 fold change (log2FC) ≥ 1.5 and false discovery rate (FDR)‐adjusted p‐value < 0.05 were used as the screening criteria. Variable Importance in Projection (VIP) score (VIP ≥ 1) was employed to identify significantly differential metabolites between the shDLST/WT and shDLST/K409R groups.

### 2.16 Metabolic Flux Analysis

First, a chemically defined tracer medium was prepared for metabolic flux analysis. This medium was formulated with glucose‐free DMEM (11966025, Gibco) supplemented with ^13^C‐labeled glucose (HY‐B0389A, MCE). Cells were evenly seeded into 100 mm culture dishes and incubated with the aforementioned tracer medium at 37°C for 24 h. Cell pellets were subsequently harvested using prechilled 80% methanol, snap‐frozen in liquid nitrogen, and then sent for metabolomic profiling at Metabo‐Profile (Shanghai, China).

### 2.17 Seahorse Assay

The Oxygen consumption rate (OCR) was measured using a Seahorse XFe96 analyzer (Agilent Technologies, Santa Clara, CA) following the protocol of the Mito Stress Test Kit (103015‐100, Agilent Technologies). Extracellular Acidification Rate (ECAR) assays were performed following the manufacturer's protocol of the Glycolysis Stress Test Kit (103020‐100, Agilent Technologies). Cells were counted and seeded into an XF96 microplate at a density of 15,000 cells/well, then cultured for 12 h. Subsequently, cells were washed and incubated in base medium (Agilent Technologies) at 37°C for 1 h. Different drug‐containing ports were supplemented with 1.5 µM oligomycin, 1.0 µM FCCP, and 0.5 µM rotenone. For ECAR measurement, cells were cultured for 12 h, after which 10 mM glucose, 1 µM oligomycin, and 50 mM 2‐DG were added to different ports of the Seahorse cartridge. The final results were normalized to the protein concentration of cells per well. Three independent replicates were set for each group.

### 2.18 ATP Content Assay

To determine ATP production levels, intracellular ATP content was measured using an Enhanced ATP Assay Kit (S0027, Beyotime) according to the manufacturer's instructions. The obtained values were normalized to protein concentration.

### 2.19 Reactive Oxygen Species (ROS) Assay

LUAD cells after different treatments were collected and incubated with DCFH‐DA (S0033S, Beyotime) at a final concentration of 10 µm in FBS‐free DMEM for 20 min at 37°C. Flow cytometry (FlowSight, Millipore, Billerica, MA) was then employed to analyze intracellular ROS levels.

### 2.20 NADP^+^/NADPH Ratio Assay

The intracellular NADP^+^ and NADPH levels in cells were detected using the Enhanced NADP^+^/NADPH Assay Kit (S0180S, Beyotime) according to the kit instructions. Cells were lysed on ice with extraction buffer, and lysates were centrifuged at 10 000×g for 10 min. A portion of the supernatant was reacted with the working solution at 27°C for 20 min, and the absorbance at 450 nm was measured to determine the total concentration of NADP^+^ and NADPH. Another portion of the supernatant was incubated at 60°C to decompose NADP^+^; after reaction with the working solution, absorbance at 450 nm was measured to obtain the concentration of NADPH alone. The NADP^+^/NADPH ratio was calculated using the standard curve and the provided formula.

### 2.21 Mitochondrial Membrane Potential (MMP) Detection

Cells were seeded in 6‐well plates and incubated overnight. After washing with PBS, cells were stained with JC‐1 working solution (J6004L, Uelandy, Suzhou, China) at 37°C for 20 min. MMP was detected via relative fluorescence ratio staining, with red/green ratio indicating mitochondrial membrane potential.

### 2.22 Mitochondrial Function Assay

Cells were incubated with Mito‐Red (M7512, Invitrogen) at a concentration of 25 nM for 15 min at 37°C in the dark, followed by staining with DAPI (E607303, BBI Life Sciences, Shanghai, China). Images were then captured and analyzed using a laser scanning confocal microscope (LSM 510 META, Carl Zeiss, Germany).

### 2.23 Mass Spectrometry Analysis

H1299‐Flag‐DLST WT cells were lysed in IP buffer supplemented with 10% protease inhibitor. Subsequently, the cell lysate was incubated overnight with Dynabeads Protein G (10004D, Invitrogen) and Flag‐tag antibody (80010‐1‐RR, Proteintech). The bead‐bound samples were then sent to Bio‐prolab Biotechnology (Wuhan, China) for mass spectrometry analysis.

### 2.24 Immunofluorescence (IF) Staining

Cells were seeded on PLS‐coated glass coverslips and incubated overnight, followed by fixation with 4% paraformaldehyde. Cells were permeabilized with 5% Triton X‐100 and blocked with 10% donkey serum at room temperature. Primary antibodies against DLST, CPT1A and TOM20 were then added and incubated overnight at 4°C. The next day, after three washes with PBS, cells were incubated with rabbit anti‐rat IgG (A‐21211, Thermo Fisher Scientific) or goat anti‐mouse IgG (A‐21212, Thermo Fisher Scientific). Nuclei were stained with DAPI solution (E607303, BBI Life Sciences). Colocalization of DLST and CPT1A in cells was observed using a confocal microscope (LSM 510 META, Carl Zeiss, Germany).

### 2.25 Glutathione S‐Transferase (GST) Pull‐Down Assay

The GST‐DLST‐WT and GST‐DLST‐K409R fusion proteins were expressed in E. coli BL21 at 37°C cultured overnight, followed by IPTG induction (0.1 mM) overnight. GST‐Tag proteins were purified using the GST‐tag protein purification kit (P2262, Beyotime). Purified GST‐fused proteins were incubated with cytoplasmic extracts containing His‐CPT1A under optimal conditions to allow interactions and their interaction was detected by Western blot.

### 2.26 Steady‐State Kinetics of DLST Succinylation Mediated by CPT1A

Recombinant His‐CPT1A‐WT or His‐CPT1A‐H473A proteins immunoprecipitated from 293T cells were co‐incubated with purified recombinant GST‐DLST protein in the presence of succinyl‐CoA at gradient concentrations (0, 0.078125, 0.15625, 0.3125, 0.625, 1.25, 2.5, 5, 10, 20, and 40 µm). Steady‐state kinetic parameters of CPT1A enzymatic activity were determined by quantifying the production of coenzyme A via colorimetric measurement of absorbance at 570 nm using the Coenzyme A DetectionKit (ab102504, Abcam, Cambridge, MA), in strict accordance with the manufacturer's protocol [26].

### 2.27 Transmission Electron Microscopy (TEM)

Cells treated with DMSO or ES‐Cu (5 nM ES+1 µM CuCl_2_) were collected and fixed with 2.5% glutaraldehyde (AWI0097, Abiver, Changsha, China), then sent to Wellbio (Changsha, China) for further processing. Images were captured and analyzed using a transmission electron microscope (JEM1400, JEOL, Tokyo, Japan).

### 2.28 Inductively Coupled Plasma Mass Spectrometry (ICP‐MS)

To determine mitochondrial copper content, H1299 shDLST/WT, H1299 shDLST/K409R, H358 shDLST/WT, and H358 shDLST/K409R cells were harvested. Mitochondria were isolated and purified using a mitochondrial extraction kit (C3601, Beyotime), and the copper levels in the purified mitochondria were subsequently measured via ICP‐MS (Puyan Biotechnology, Beijing).

### 2.29 Virtual Screening of Small‐Molecule Compounds Targeting DLST K409

Virtual screening of small‐molecule compounds targeting the DLST K409 was conducted by MCE. Briefly, the three‐dimensional structure of human DLST (PDB ID: 6H05) was downloaded from the RCSB PDB database and processed for hydrogen addition using the Protein Preparation Wizard module. A total of 1,541 compounds were initially screened from the compound library HY‐L0076V (containing 10 480 compounds) using DataWarrior software. These compounds were further processed for hydrogen addition and energy minimization via the LigPrep Module of Schrödinger software before virtual docking. A higher absolute value of the predicted docking score indicated stronger binding affinity between the compound and the protein. The top three compounds with the highest absolute docking scores were purchased from MCE as candidates for subsequent experimental validation.

### 2.30 Cellular Thermal Shift Assay (CETSA)

To evaluate the binding efficiency of SI409‐1 with DLST, H1299 cells were treated with 100 µM SI409‐1/DMSO for 24 h, collected, and evenly divided into six samples, each of which was heated at different temperatures for 10 min. Protein levels were subsequently analyzed by Western blotting to assess the stability of DLST protein under thermal conditions.

### 2.31 Patient‐Derived Organoid (PDO) Models

Fresh clinical lung adenocarcinoma tissues were thoroughly rinsed with sterile PBS and cut into small pieces of 1×1×1mm^3^. Clinicopathological data of patients are shown in Table S5. The tissue fragments were digested with type IV collagenase in a thermostatted shaking bath at 37°C for 1 h. Cell pellets were collected by centrifugation and resuspended in prechilled organoid culture medium (Mogengel, MA‐0807T002LP). The cell suspension was mixed with Matrigel (Mogengel, 827035) at a volume ratio of 3:7, and the mixture was seeded into 24‐well plates. After gel solidification, additional organoid medium was added to each well. Organoids were cultured in a humidified incubator at 37°C with 5% CO_2_. PDOs at passages 3–6 were uniformly used for drug intervention assays.

### 2.32 Drug‐Sensitivity Analysis

Organoids were resuspended in Matrigel and embedded as a suspension in 96‐well plates (500 cells per well, with Matrigel added at a 1:1 volume ratio to the cell suspension). After 1 day incubation, the medium was replaced with fresh medium containing drugs at different concentrations, and the organoids were cultured for an additional 5 days. At the experimental endpoint, the number of viable cells was measured using the CellTiter‐Glo assay kit (G7572, Promega) according to the manufacturer's instructions.

### 2.33 Molecular Docking Analysis

Download the PDB files of DLST‐WT and DLST‐K409R from the Swiss database, and obtain the SDF file of lipoyl‐AMP (PubChem CID: 23724672) from PubChem (https://pubchem.ncbi.nlm.nih.gov/). Then perform molecular docking using the CB‐Dock2 tool (http://cadd.labshare.cn/cb‐dock2/). A higher Vina Score indicates a weaker binding capacity.

### 2.34 Bioinformatics Analysis

The Uniport database (https://www.uniprot.org/) was used to analyze the conservation of succinylation sites in DLST. The Clinical Proteomic Tumor Analysis Consortium (CPTAC) database was employed to investigate the expression levels of DLST and CPT1A in LUAD. Survival curves of LUAD patients with high and low DLST expression were analyzed using the TCGA‐LUAD public dataset. The GDSC dataset was utilized to investigate the sensitivity of CPT1A expression to standard LUAD treatment regimens. Kaplan‐Meier (KM) Plotter (https://kmplot.com/analysis/) was utilized to generate survival curves for LUAD patients stratified by CPT1A expression levels.

### 2.35 Statistical Analysis

Statistical analyses were performed using GraphPad Prism 8 software. Quantitative data from all experiments are presented as the mean ± standard deviation (SD). The statistical analysis was performed by a two‐tailed unpaired Student's t test, two‐way analysis of variance (ANOVA) followed by the multiple comparisons Bonferroni test. The correlations were calculated using the parametric two‐tailed Pearson correlation test. Statistical significance was defined as p < 0.05. Levels of significance were indicated as follows: *p <0.05, **p < 0.01, ***p < 0.001, and ns for non‐significant.

## 3 Results

### 3.1 DLST is Highly Succinylated on K409 in LUAD

**FIGURE 1:**
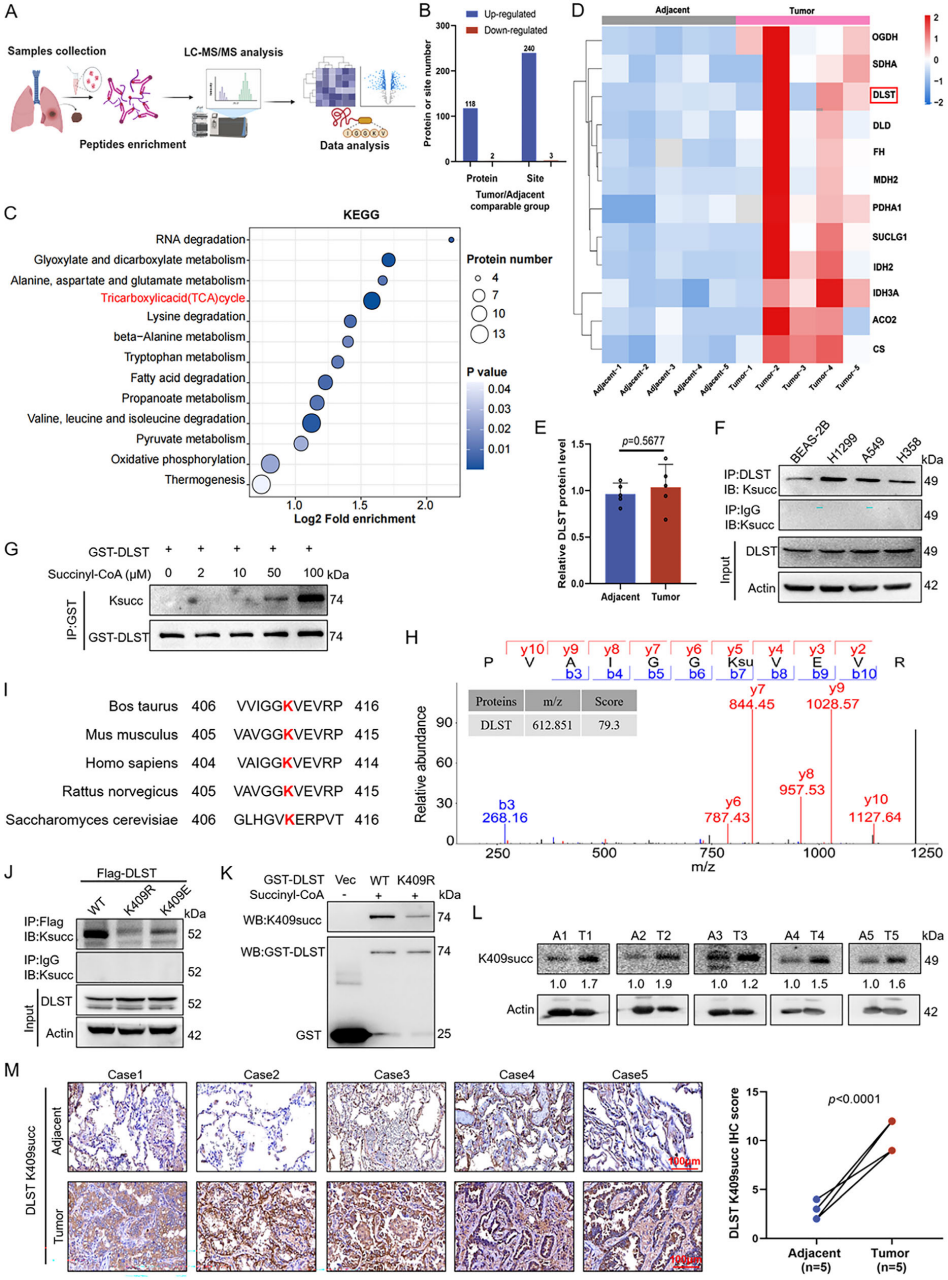
DLST is highly succinylated on K409 in LUAD. (A) Schematic diagram of the quantitative proteomic and succinylation proteomics workflow based on 5 pairs of lung adenocarcinoma and adjacent normal tissue samples. (B) Number of succinylated proteins and sites identified by 4D label‐free lysine succinylation proteomics mass spectrometry analysis. (C) KEGG pathway enrichment analysis of succinylated proteins. (D) Heatmap analysis of differentially succinylated proteins involved in the TCA cycle (|Fold change| >1.5). (E) Proteomic data analysis of DLST protein expression in LUAD tissues and adjacent normal tissues (*n* = 5). (F) IP assay to detect DLST succinylation in BEAS‐2B, H1299, A549, and H358 cells. (G) IP assay to detect DLST succinylation changes with the concentration of succinyl‐CoA. (H) DLST succinylation at the K409 site from 4D label‐free lysine succinylation proteomics mass spectrometry analysis. (I) The sequences around DLST K409 from different species were aligned. (J) After transfecting Flag‐DLST, Flag‐DLST‐K409R, and Flag‐DLST‐K409E plasmids into 293T cells for 48 h, an IP assay was performed using a DLST K409succ antibody to detect the succinylation levels of DLST WT and its mutants. (K) Purified GST‐DLST‐WT and GST‐DLST‐K409R proteins were incubated with succinyl‐CoA (50 µM) at 37°C for 30 min in vitro, and immunoblot analysis was performed using the corresponding antibodies. (L) Western Blot to detect DLST K409succ in LUAD tissues and adjacent normal tissues. (M) IHC staining to detect DLST K409succ in LUAD tissues and adjacent normal tissues (*n* = 5). Statistical significance was determined by a two‐tailed unpaired Student's *t*‐test for (E, M).

To explore the global role of protein succinylation in the malignant progression of LUAD, we collected 5 pairs of primary LUAD tumor tissues and matched adjacent normal tissues from patients. Then quantitative proteomic and succinylation proteomics were performed, with the workflow illustrated in Figure 1A. Pearson correlation analysis demonstrated excellent reproducibility of the data (Figure S1A). Principal component analysis (PCA) showed a significant separation between tumor and adjacent tissue samples (Figure S1B). Succinylation proteomic analysis quantified 120 proteins and 243 succinylation sites, compared with normal tissues, 118 proteins and 240 sites exhibited upregulated succinylation, and 2 proteins and 3 sites showed downregulated succinylation in tumor tissues (Figure 1B). The modificomics volcano plot showed the distribution of differentially succinylated proteins (Figure S1C). Further, the results of KEGG enrichment analysis revealed that differentially succinylated proteins were enriched in a wide range of cellular metabolic processes, with the most prominent enrichment in the TCA cycle (Figure 1C). Meanwhile, subcellular localization analysis showed that 62.07% of the succinylated proteins were localized to mitochondria (Figure S1D), which suggests a close association between succinylation and the regulation of mitochondrial metabolism. The heatmap of proteins enriched in the TCA cycle demonstrated that the succinylation of proteins such as succinate dehydrogenase complex subunit A (SDHA), oxoglutarate dehydrogenase (OGDH), and DLST was significantly upregulated in LUAD tissues (Figure 1D). Due to the succinylation of SDHA and OGDH in tumors, which has been previously reported [27, 28], the role and mechanism of DLST succinylation in malignant tumors remain unelucidated. Therefore, DLST was selected as the research target in this study.

The proteomic data showed no significant difference in DLST protein expression between LUAD and adjacent normal tissues (Figure 1E). Further analysis of data from the CPTAC public database displayed consistent results (Figure S1E), and KM‐plotter curves revealed no significant correlation between DLST expression and the prognosis of LUAD patients (Figure S1F). Meanwhile, IP experiments demonstrated that DLST was highly succinylation in LUAD cells compared to BEAS‐2B cells (Figure 1F). To determine whether DLST is succinylated in vitro, we incubated purified recombinant GST‐DLST with succinyl‐CoA and detected DLST succinylation, which was enhanced by increased concentrations of succinyl‐CoA (Figure 1G). Collectively, these results suggest that DLST succinylation rather than its expression may play a critical role in the malignant progression of LUAD. The mass spectrometry analysis revealed that DLST K409 is succinylated in LUAD, and the DLST K409succ in LUAD tissues is 7.68‐fold higher than that in adjacent normal tissues (Figure 1H, Figure S2A). A UniProt database analysis showed that K409 is highly conserved across species (Figure 1I). The mutation of lysine (K) to arginine (R) could mimic the desuccinylated state, and the mutation of K to glutamate (E) could mimic the succinylation modification state [29]. We subsequently constructed Flag‐DLST‐K409R and Flag‐DLST‐K409E plasmids to simulate succinylate deficiency and succinylate status, respectively (Figure S2B). After transfection into 293T cells, IP experiments data demonstrated significantly reduced succinylated levels of DLST after K409R mutation, while K409E mutation increased succinylated levels (Figure 1J). Further, we developed a specific DLST K409 polyclonal antibody (DLST‐K409Succ), Western blot and ELISA analyses revealed stronger signals for modified peptides compared to unmodified ones, indicating excellent specificity (Figure S2C–E). We incubated purified recombinant GST‐DLST‐WT or GST‐DLST‐K409R with succinyl‐CoA in vitro, finding that DLST K409R could significantly reduce succinylation of DLST compared to the WT (Figure 1K). These experiments further demonstrated that K409 is the primary succinylation site of DLST. In addition, Western blot and IHC analysis on clinical tissue samples also showed significantly higher DLST K409succ in LUAD than in adjacent normal tissues (Figure 1L,M). The above results indicate that DLST is highly succinylated at K409 in LUAD.

### 3.2 DLST K409succ Promotes the Malignant Progression of LUAD

**FIGURE 2:**
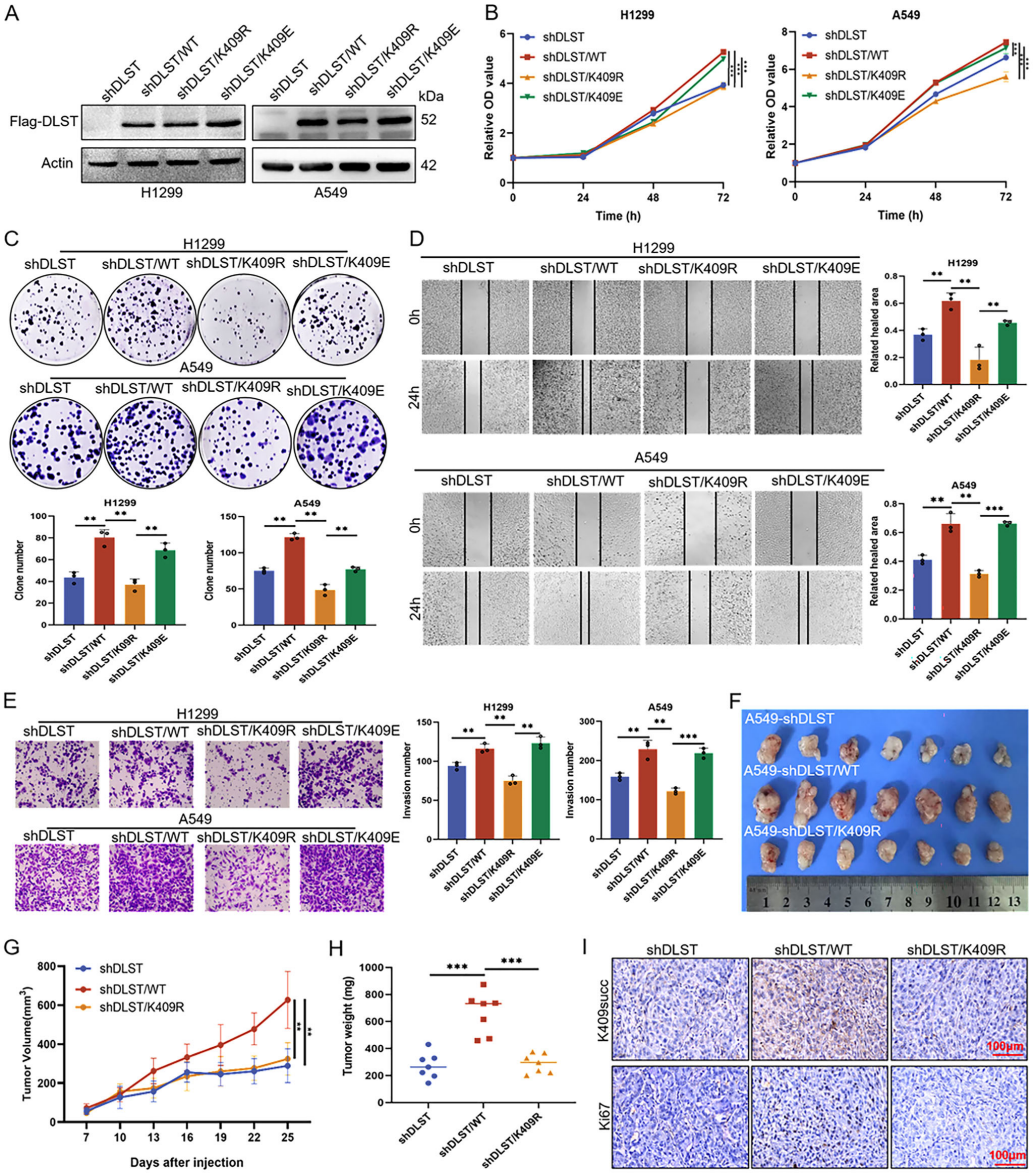
DLST K409succ promotes the malignant progression of LUAD. (A) The expression of DLST in stable cell lines H1299‐shDLST, H1299‐shDLST/WT, H1299‐shDLST/K409R, H1299‐shDLST/K409E, A549‐shDLST, A549‐shDLST/WT, A549‐shDLST/K409R, and A549‐shDLST/K409E was detected by Western Blot. Using the above cells, (B) CCK‐8 and (C) colony formation assays were used to detect cell proliferation, (D) Wound‐healing, and (E) Transwell assay were used to detect cell migration and invasion (*n* = 3). After 5 × 10^6^ A549‐shDLST, A549‐shDLST/WT, and A549‐shDLST/K409R stable cells were injected subcutaneously into 5‐week‐old nude mice (*n* = 7), the tumor growth (F), tumor volume (G), and tumor weight (H) were monitored. (I) Representative images of IHC staining of DLST K409succ and Ki‐67 in xenograft tumor tissues. Data were shown as the mean ± SD of at least three independent experiments. **p < 0.01, ***p < 0.001. Statistical significance was determined by two‐tailed unpaired Student's *t*‐test (C, D, E, H) and the two‐way ANOVA (B, G).

To investigate the effect of DLST K409succ on the biological functions of LUAD cells, we first established stable H1299 and A549 cell lines with DLST knockdown (Figure S3A). CCK‐8 and colony formation assays demonstrated that shDLST significantly reduced the proliferation of LUAD cells (Figure S3B,C). Wound‐healing and transwell assays revealed that shDLST impaired the migration and invasion abilities of LUAD cells (Figure S3D,E). Subsequently, we further established stable cell lines expressing Flag‐DLST‐WT, Flag‐DLST‐K409R, or Flag‐DLST‐K409E in H1299‐shDLST or A549‐shDLST cells, respectively (Figure 2A). CCK‐8 and colony formation assays demonstrated that compared with shDLST, shDLST/WT and shDLST/K409E promoted cell proliferation, while the DLST K409R mutant significantly inhibited the proliferation of LUAD cells (Figure 2B,C). Wound‐healing assays and Transwell assays showed that shDLST/WT and shDLST/K409E enhanced the migration and invasion abilities of LUAD cells, whereas the DLST K409R mutant suppressed the migration and invasion of tumor cells (Figure 2D,E). We further employed a xenograft mouse model to evaluate the tumor‐promoting effect of DLST K409succ in vivo. The results showed that both the tumor volume and weight in the DLST WT group were significantly higher than those in the shDLST group, whereas DLST K409R significantly suppressed tumor growth compared with the DLST WT group (Figure 2F–H). IHC results demonstrated that the level of DLST K409succ and Ki67 was decreased in the DLST K409R cells group compared with the DLST WT group (Figure 2I). In summary, these data confirm that DLST K409succ promotes the malignant progression of LUAD.

### 3.3 DLST K409succ Enhances the Enzymatic Activity and Mitochondrial Metabolism and Function in LUAD Cells

**FIGURE 3:**
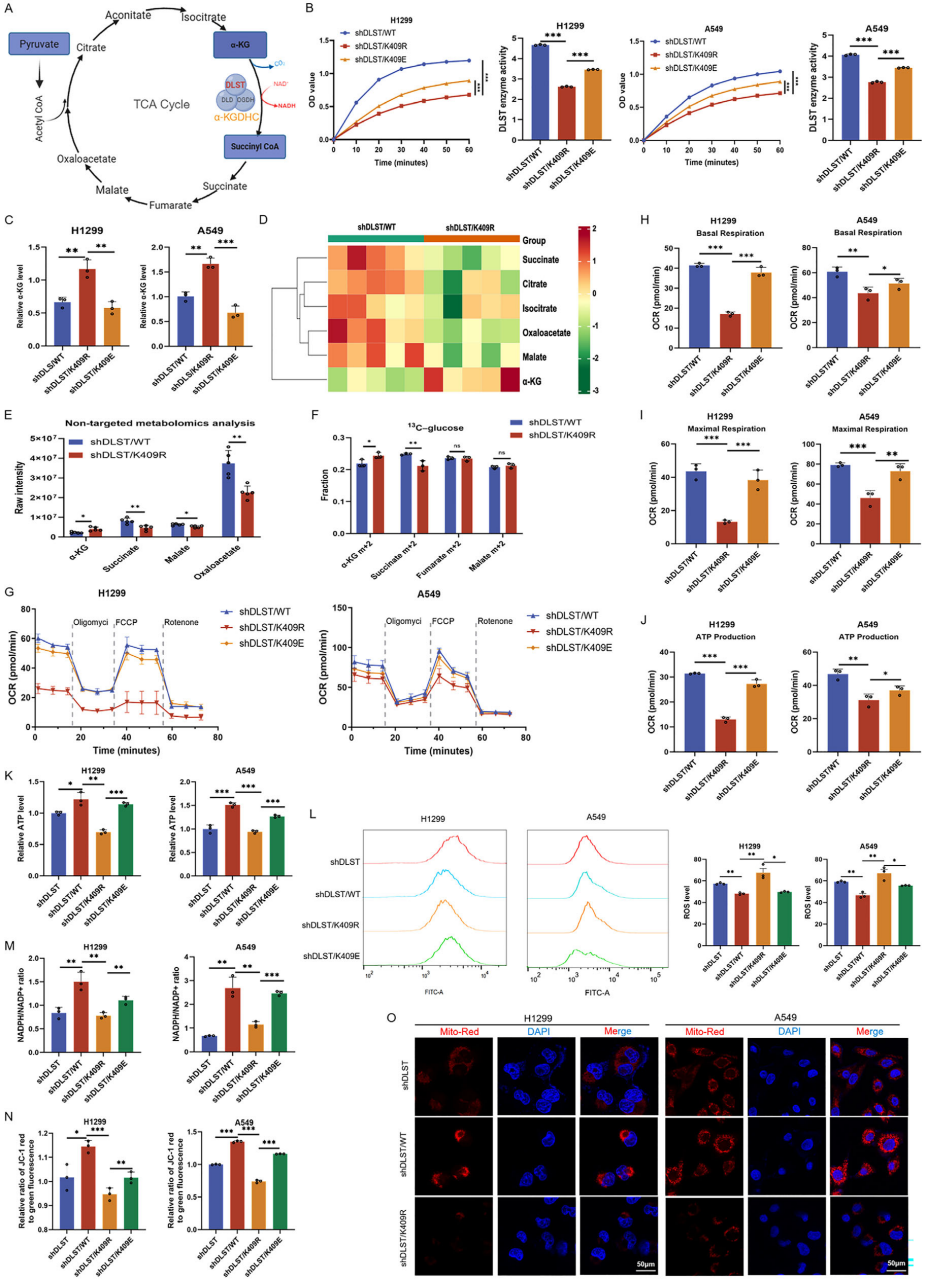
DLST K409succ enhances the enzymatic activity and mitochondrial metabolism and function in LUAD cells. (A) Schematic overview of DLST and α‐KGDHC enzymes involved in the TCA cycle. In the stable cell lines H1299‐shDLST/WT, H1299‐shDLST/K409R, H1299‐shDLST/K409E, A549‐shDLST/WT, A549‐shDLST/K409R, and A549‐shDLST/K409E, (B) α‐KGDHC enzymatic activity was detected using an α‐KGDHC activity assay kit (*n* = 3). (C) α‐KG content was detected using an α‐KG assay kit (*n* = 3). (D) Cluster analysis of TCA cycle metabolites and (E) Changes in upstream and downstream products of DLST in A549‐shDLST/WT and A549‐shDLST/K409R cells through non‐targeted metabolomics analysis. (F) Metabolomic flux analysis using ^1^ ^3^C‐glucose tracing between A549‐shDLST/WT and A549 DLST/K409R cells (*n* = 3). In the stable cell lines H1299‐shDLST, H1299‐shDLST/WT, H1299‐shDLST/K409R, H1299‐shDLST/K409E, A549‐shDLST, A549‐shDLST/WT, A549‐shDLST/K409R, and A549‐shDLST/K409E, (G) Mitochondrial respiration was detected using the OCR assay (*n* = 3). (H‐J) Comparison of basal respiratory rate (H), maximal respiratory rate (I), and ATP production rate (J). (K) Detection of ATP content using an ATP content assay kit (*n* = 3). (L) Flow cytometric analysis of intracellular ROS levels (*n* = 3). (M) Detection of the NADPH/NADP^+^ ratio using an NADPH/NADP^+^ assay kit (*n* = 3). (N) Detection of MMP in LUAD cells after incubation with JC‐1 probe for 30 min (*n* = 3). (O) Confocal imaging showing mitochondria labeled with Mito‐red (red) and localization of cell nuclei stained with DAPI (blue). Data were shown as the mean ± SD of at least three independent experiments. *p < 0.05, **p < 0.01, ***p < 0.001. Statistical significance was determined by two‐tailed unpaired Student's t‐test (C, E, K, L, M, N) and the two‐way ANOVA (B, H, I, J).

Mitochondria serve as the central organelles for metabolic activities, with DLST being an important component of the key rate‐limiting enzymes α‐KGDHC in the TCA cycle (Figure 3A). To determine whether DLST K409succ regulates its enzyme activity, using the α‐KGDHC enzyme activity assay kit, the data showed that DLST K409R significantly reduced the enzyme activity (Figure 3B). Meanwhile, substrate α‐KG levels assay revealed a marked increase in enzymatic α‐KG concentration following K409R mutation (Figure 3C). Through non‐targeted metabolomics analysis of A549‐shDLST/WT and A549‐shDLST/K409R cells, PCA demonstrated good reproducibility of samples (Figure S4A). Compared to A549‐shDLST/WT cells, A549‐shDLST/K409R cells exhibited 133 upregulated and 960 downregulated metabolites (Figure S4B). Importantly, through non‐targeted metabolomics analysis, it was found that in A549‐shDLST/K409R cells, the substrate α‐KG of DLST was significantly increased, while the product succinate was significantly lower in shDLST/K409R than in shDLST/WT cells (Figure 3D,E). To explore the effects of DLST K409 succinylation on mitochondrial metabolism in LUAD cells at the dynamic flux level, we performed metabolomic flux analysis using ^1^ ^3^C‐glucose tracing. The results revealed that the abundance of m+2 α‐KG, the substrate of the DLST‐catalyzed reaction, was markedly increased, while the level of its product m+2 succinate was significantly decreased in DLST‐K409R cells (Figure 3F). This result further demonstrates that DLST K409 succinylation directly enhances the OXPHOS pathway in LUAD cells. Further, we used the OCR assay to assess whether DLST K409succ affects mitochondrial respiration. The results showed that shDLST/K409R impaired mitochondrial respiration compared to shDLST/WT, while shDLST/K409E rescued the compromised respiration and enhanced mitochondrial activity (Figure 3G–J). Compared with the shDLST group, the ATP content in LUAD cells of the shDLST/WT group was increased, whereas compared with the shDLST/WT group and shDLST/K409E group, the intracellular ATP content in LUAD cells of the shDLST/K409R group was significantly decreased (Figure 3K). In addition, ECAR data showed that the K409R mutant did not affect glycolysis levels or ATP production via the glycolytic pathway in LUAD cells (Figure S4C,D). These results indicate that DLST succinylation promotes malignant progression of LUAD predominantly through the OXPHOS pathway.

To verify that metabolic reprogramming mediated by DLST K409 succinylation acts as a driver of malignant phenotypes in LUAD cells, we performed rescue experiments by supplementing metabolic intermediates. Desuccinylation of DLST K409 impairs the metabolic flow from α‐KG to succinyl‐CoA and subsequently to succinate. Notably, methylsuccinate can enter the TCA cycle and functionally substitute for succinate [20, 30, 31]. Therefore, we added cell‐permeable dimethyl succinate at a concentration of 5 mM to the culture medium of H1299‐shDLST/WT, H1299‐shDLST/K409R, A549‐shDLST/WT, and A549‐shDLST/K409R cells. Functional rescue assays, including CCK‐8, colony formation, and transwell assays, were then conducted. The results demonstrated that succinate supplementation significantly rescued the defects in cell proliferation and invasion caused by the DLST K409R mutation in LUAD cells (Figure S4E–G). Furthermore, DLST K409R increased ROS levels and decreased the NADPH/NADP+ ratio in LUAD cells (Figure 3L,M). Subsequent analysis of MMP revealed significantly lower MMP in shDLST/K409R cells compared to the shDLST/WT group (Figure 3N). Confocal imaging analysis using Mito‐Red further demonstrated that shDLST/WT cells exhibited higher mitochondrial activity, whereas shDLST/K409R effectively reduced it (Figure 3O). From these results, we conclude that DLST K409succ enhances the enzymatic activity and mitochondrial metabolism and function in LUAD cells.

### 3.4 CPT1A Mediates the DLST K409succ

**FIGURE 4:**
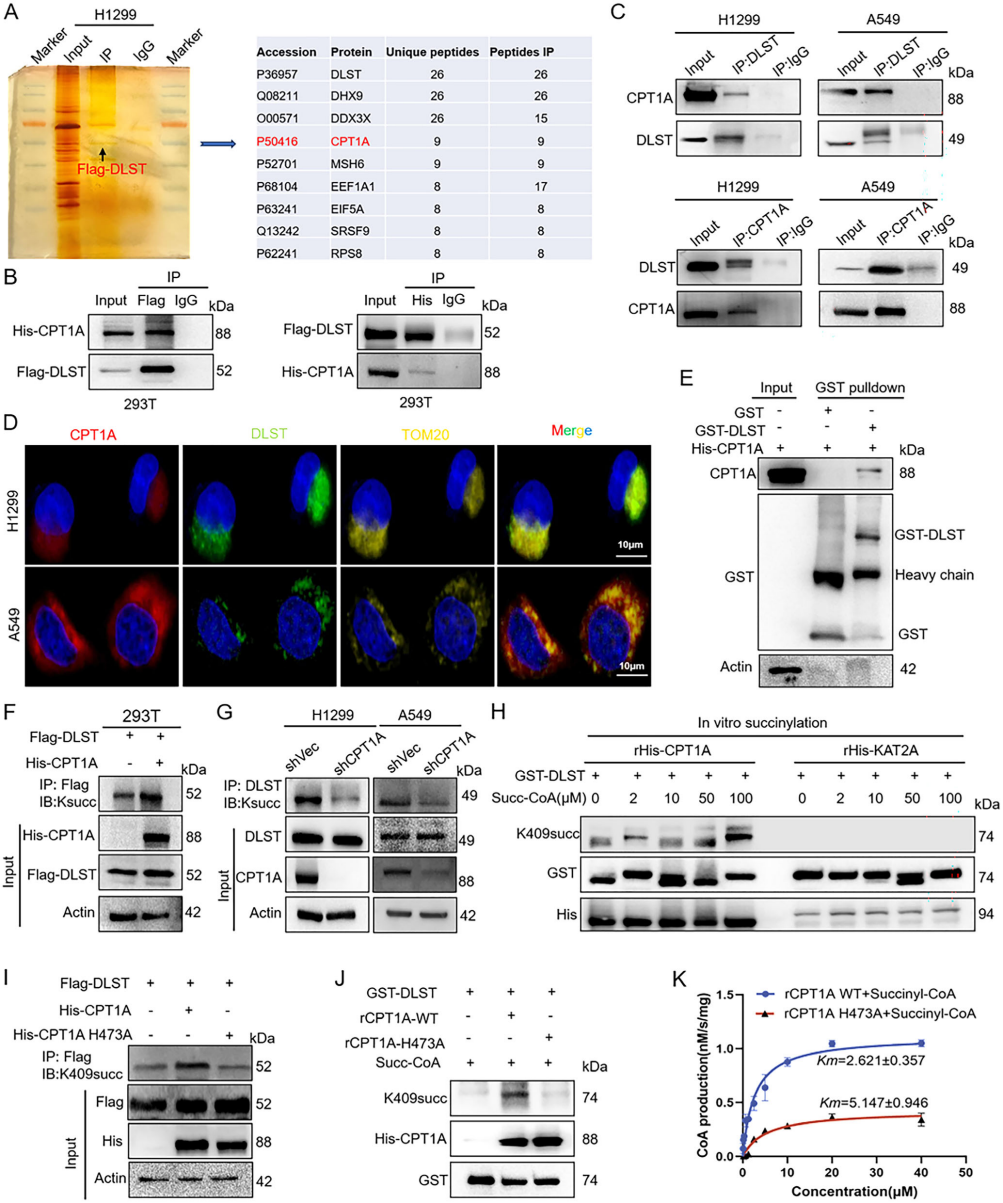
CPT1A mediates the DLST K409succ. (A) Left, silver staining of IP proteins enriched with DLST antibody in H1299 cell; Right, preliminary mass spectrometry screening results, with red highlights succinyltransferase. (B) After co‐transfected His‐CPT1A and Flag‐DLST plasmid in 293T cells for 48 h, IP analysis was performed to detect the interaction between CPT1A and DLST using Flag‐Tag and His‐Tag antibody. (C) Endogenous IP experiments to detect the interaction between DLST and CPT1A in LUAD cells. (D) Confocal microscopy to detect the colocalization of DLST and CPT1A in LUAD cells. TOM20 was utilized as a mitochondrial marker and DAPI for nuclear staining. (E) After 48 h of exogenous expression of His‐CPT1A in 293T cells, IP was performed using a GST antibody to detect the interaction between GST, GST‐DLST purified proteins and CPT1A. (F) After 48 h of transient transfection of His‐CPT1A and Flag‐DLST plasmids into 293T cells, respectively, IP and Western Blot were used to detect the succinylation level of DLST.(G) Knockdown CPT1A in H1299 and A549 cells, respectively, and the succinylation level of DLST was detected using IP and Western Blot. (H) In the in vitro succinylation reaction system, the recombinant protein CPT1A (rHis‐CPT1A) ‐added group and the recombinant protein KAT2A(rHis‐KAT2A)‐added group were established, with a gradient of succinyl‐CoA concentrations (0, 2, 10, 50, and 100 µM) included in each group. The DLST‐K409succ was subsequently detected by Western blot analysis. (I) After transfecting 293T cells with Flag‐DLST, Flag‐DLST+His‐CPT1A, and Flag‐DLST+His‐CPT1A H473A plasmids, respectively, for 48 h, an IP assay was performed to analyze the effect of the CPT1A mutant on the succinylation of DLST. (J) In the in vitro succinylation system, GST‐DLST was incubated with recombinant CPT1A (rCPT1A) or the rCPT1A H473A mutant at 37°C for 30 min. Western blot analysis was then performed to assess the in vitro succinylation of DLST. (K) The steady‐state kinetics of CPT1A‐mediated DLST succinylation modifications.

To identify the succinyltransferase mediating DLST succinylation, we performed mass spectrometry analysis, and the results showed that CPT1A interacted with DLST (Figure 4A). Further, exogenous and endogenous IP assays confirmed the interaction between CPT1A and DLST (Figure 4B,C). IF staining indicated substantially increased colocalization of CPT1A and DLST, and both proteins were predominantly localized to mitochondria (Figure 4D). GST‐pull down assays also confirmed the direct binding between the CPT1A and DLST (Figure 4E). Moreover, overexpression of CPT1A significantly increased DLST succinylation, while stable knockdown of CPT1A in H1299 and A549 cells decreased DLST succinylation (Figure 4F,G). Meanwhile, to further verify that CPT1A acts as the primary succinyltransferase responsible for DLST K409 succinylation, we conducted in vitro succinylation assays using recombinant proteins and compared the effects of CPT1A and the well‐known succinyltransferase KAT2A. The results demonstrated that compared with KAT2A, CPT1A significantly promoted the DLST K409 succinylation in vitro (Figure 4H). Next, we investigated whether CPT1A specifically mediates DLST K409succ by using CPT1A active site mutation plasmid His‐CPT1A H473A [32]. The results demonstrated that wild‐type CPT1A increased DLST K409succ, whereas CPT1A mutants reduced it (Figure 4I).

After in vitro expression and purification of His‐CPT1A and His‐CPT1A H473A, in vitro succinylation assays were performed using recombinant GST‐DLST as the substrate and succinyl‐CoA as the donor. The results revealed that CPT1A directly catalyzes succinylation of DLST K409. In contrast, the enzymatically inactive CPT1A H473A mutant markedly reduced DLST K409 succinylation (Figure 4J). Furthermore, following methods reported in the literature [17, 26, 33], the reaction kinetics (*Vmax*) of CPT1A‐catalyzed reactions were determined under substrate saturation conditions, and the *Km* for the succinylation of DLST by recombinant CPT1A in the presence of succinyl‐CoA was calculated. The results showed that the *Km* value of CPT1A wild type for succinyl‐CoA was lower than that of the H473A mutant, indicating higher binding affinity of the wild type for succinyl‐CoA compared to the mutant (Figure 4K). These data indicated that CPT1A functions as the succinyltransferase mediating DLST K409succ.

### 3.5 CPT1A‐Mediated DLST K409succ Promotes Malignant Progression of LUAD

**FIGURE 5:**
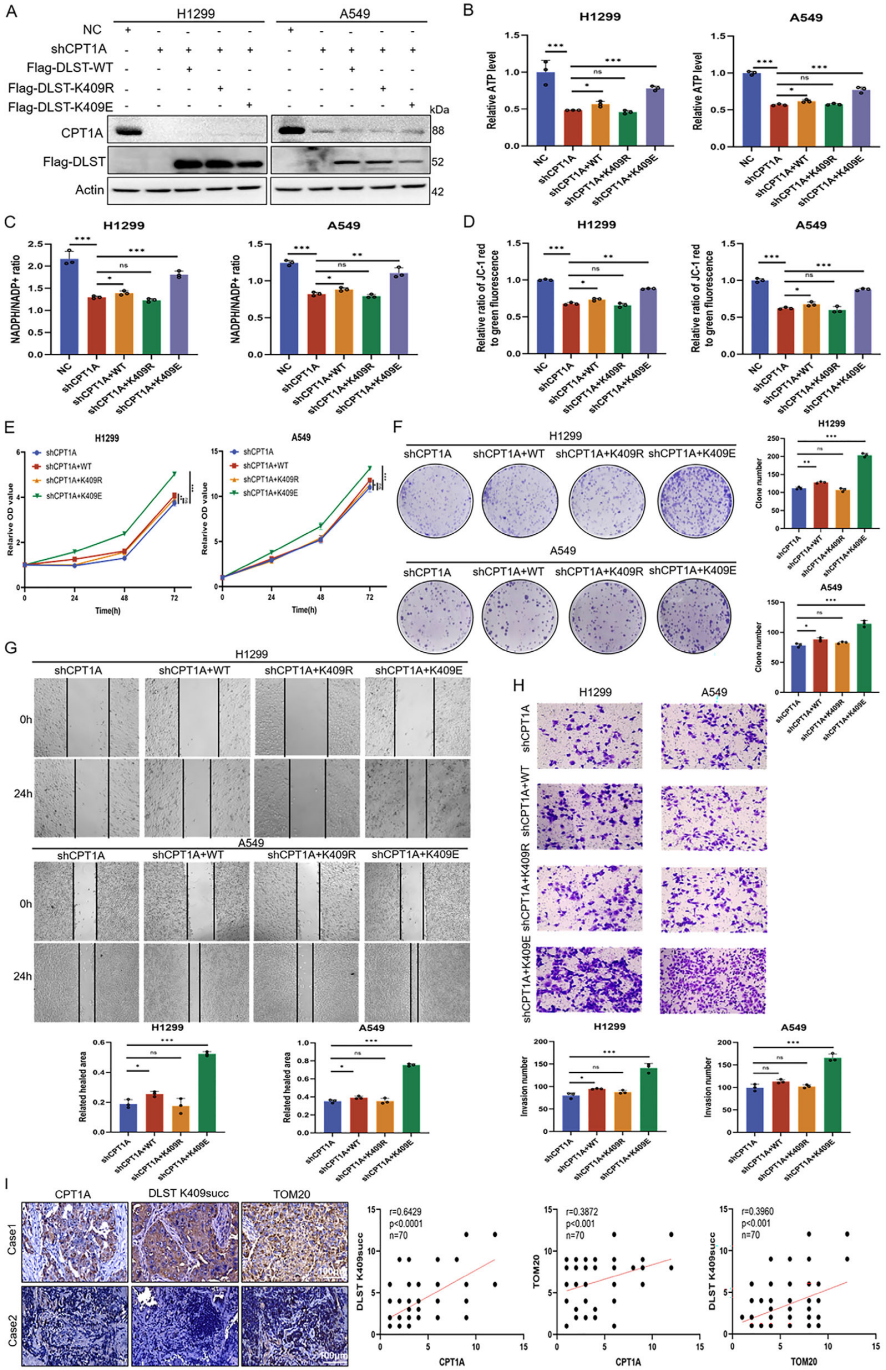
CPT1A‐mediated DLST K409succ promotes LUAD malignant progression. After stable knockdown of CPT1A in H1299 and A549 cells, lentiviral vectors expressing DLST WT, DLST K409R, and DLST K409E were transfected using a lentiviral system to establish stable cell lines. (A) The expression of CPT1A and Flag‐DLST was detected using a Western blot. (B) The ATP content was measured using an ATP content assay kit (*n* = 3). (C) The NADPH/NADP+ ratio was measured using an NADPH/NADP^+^ assay kit (*n* = 3). (D) Detection of MMP in LUAD cells after incubation with JC‐1 probe for 30 min (*n* = 3). (E–H) Proliferation capacity (E), colony formation capacity (F), migration capacity (G), and invasion capacity (H) (*n* = 3). (I) Representative images of IHC staining of CPT1A, DLST K409succ, and TOM20 in xenograft tumor tissues. Data were shown as the mean ± SD of at least three independent experiments. *p < 0.05, **p < 0.01, ***p < 0.001. Statistical significance was determined by two‐tailed unpaired Student's *t*‐test (B–D, F, G, H), two‐way ANOVA (E), and correlations were calculated using the parametric two‐tailed Pearson correlation test (I).

Currently, multiple papers have reported an increased expression of CPT1A in clinical lung cancer samples [34, 35, 36, 37]. However, we analyzed CPT1A expression in LUAD samples from the CPTAC database, and found no significant difference in CPT1A levels between tumor and normal tissues (Figure S5A). Further, Kaplan–Meier survival analysis based on the TCGA‐LUAD dataset revealed that high CPT1A expression was associated with poor patient prognosis (Figure S5B). Meanwhile, we examined CPT1A protein levels in the normal lung epithelial cell line BEAS‐2B and LUAD cell lines H1299, A549, H358 and PC9 via Western blot. The data showed that CPT1A was markedly upregulated in LUAD cells (Figure S5C). To investigate whether CPT1A affects LUAD cells through regulating DLST K409succ, after knocking down CPT1A in H1299 and A549 cells, we further established stable cell lines overexpressing DLST WT, DLST K409R or DLST K409E (Figure 5A). CCK‐8 and colony formation assays showed that CPT1A knockdown inhibited LUAD cell proliferation (Figure S5D,E). Similarly, wound‐healing and transwell assay results demonstrated that CPT1A knockdown suppressed LUAD cell migration and invasion (Figure S5F,G). To further explore the potential clinical implications of CPT1A in LUAD chemotherapy and targeted therapy, we analyzed the correlation between CPT1A expression and drug sensitivity using the GDSC database. CPT1A expression was significantly negatively correlated with cellular sensitivity to osimertinib (*r* = −0.279, *p* = 1.17e−10, FDR = 1.55e−9) and KRAS G12C Inhibitor‐12 (*r* = −0.157, *p* = 3.57e−4, FDR = 0.001). In contrast, no significant association was observed between CPT1A level and cisplatin sensitivity (*r* = 0.027, *p* = 0.543, FDR = 0.615) (Figure S5H).

Further, it can be found that CPT1A knockdown significantly reduced ATP generation, NADPH/NADP+ ratio, and MMP in LUAD cells, and DLST WT partially restored these effects, while DLST K409R did not, but DLST K409E obviously restored these effects (Figure 5B–D). Furthermore, the CCK‐8 and colony formation assays showed that although DLST WT had some rescued effect, DLST K409E could significantly reverse shCPT1A‐induced inhibition of malignant progression of LUAD cells, while DLST K409R failed to achieve this effect (Figure 5E,F). And wound‐healing and transwell assay results revealed that DLST WT partially rescued effect and DLST K409E rescued the shCPT1A‐induced suppression of cell migration and invasion, while DLST K409R failed to do so (Figure 5G,H). Finally, we performed IHC staining on 70 LUAD clinical samples. The results showed a positive correlation between CPT1A, DLST K409succ, and TOM20, among which TOM20 is a mitochondrial protein transport regulator that can serve as a biomarker reflecting mitochondrial activity (Figure 5I) [38, 39]. Based on IHC data and clinical information, we conducted correlation analyses. DLST K409succ expression showed an upward trend in the EGFR‐mut group, though the difference was not statistically significant. This preliminarily indicates that this pathway may be involved in EGFR‐mutant LUAD, which needs to be verified using a larger cohort (Figure S5I). Taken together, these findings indicate that CPT1A enhances mitochondrial metabolism and LUAD malignant progression by promoting DLST K409succ.

### 3.6 DLST K409succ Enhances the Cuproptosis Resistance by Inhibiting Its Lipoylation

**FIGURE 6:**
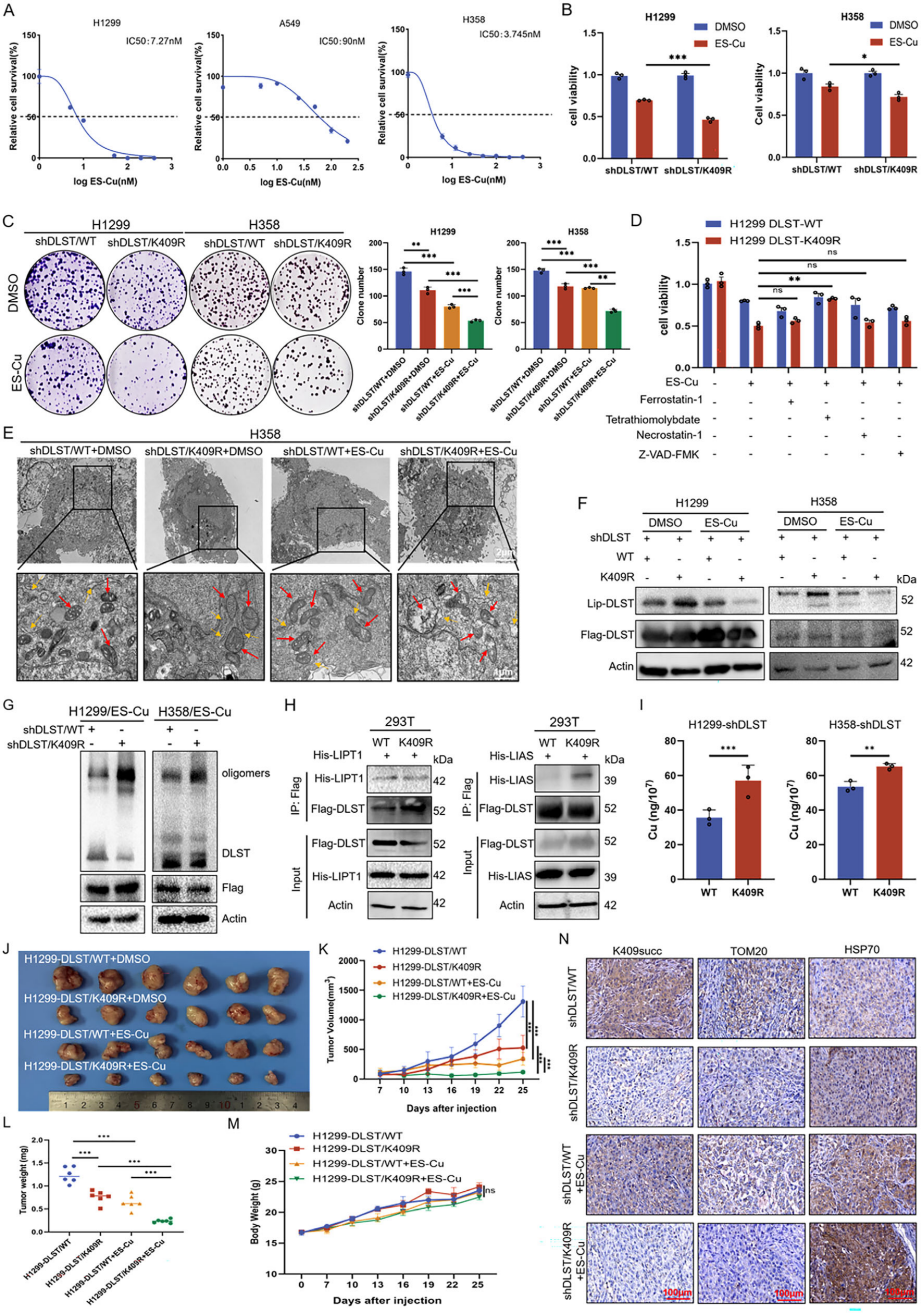
DLST K409succ enhances the cuproptosis resistance by inhibiting its lipoylation. (A) H1299, A549, and H358 cells were treated with the ES‐Cu using a gradient concentration. The IC_50_ was calculated by CCK‐8 assay. (B, C) Stable cells H1299‐shDLST/WT, H1299‐shDLST/K409R, H358‐shDLST/WT, and H358‐shDLST/K409R were treated with ES‐Cu (5 nM ES+1 µM CuCl_2_). (B) CCK‐8 and (C) colony formation assays were used to detect cell viability (*n* = 3). (D) The viability of cells pretreated overnight with 10 µM ferrostatin‐1, 10 µM TTM, 10 µM necrostatin‐1, and 10 µM Z‐VAD‐FMK and then treated with ES‐Cu (*n* = 3). (E) Morphological changes of cells after ES‐Cu treatment were observed by TEM (Red arrow, mitochondria; yellow arrow, endoplasmic reticulum). (F) Western blot was performed to detect the DLST lipoylation using an anti‐lipoic acid antibody. (G) The oligomerization of DLST and DLAT in DLST WT and K409R mutant cells after ES‐Cu treatment was detected using native PAGE. (H) 48 h after transient co‐transfection of His‐LIPT1 or His‐LIAS with Flag‐DLST‐WT or Flag‐DLST‐K409R plasmids into 293T cells, IP and Western blot analysis were performed to examine the interactions of DLST/WT and DLST/K409R with LIPT1 and LIAS. (I) ICP‐MS was used to detect mitochondrial copper ion content in H1299‐shDLST/WT, H1299‐shDLST/K409R, A549‐shDLST/WT, and A549‐shDLST/K409R cells. 5 × 10^6^ of H1299‐shDLST/WT or H1299‐shDLST/K409R cells were subcutaneously injected into nude mice and were treated with DMSO or ES‐Cu (ES:5 mg/kg + CuCl_2_: 0.06 mg/kg). The tumor growth (J), tumor volume (K), and tumor weight (L) were monitored (*n* = 6). (M) Changes in mouse body weight during the administration period (*n* = 6). (N) Representative images of IHC staining of DLST K409succ, TOM20, and HSP70 in xenograft tumor tissues. Data were shown as the mean ± SD of at least three independent experiments. ns, no significant difference. *p < 0.05, **p < 0.01, ***p < 0.001. Statistical significance was determined by two‐tailed unpaired Student's *t*‐test (C, L, N) and the two‐way ANOVA (B, D, K, M).

Cuproptosis is a novel copper‐dependent form of cell death, and it occurs via copper binding to lipoylated components such as Dihydrolipoamide S‐acetyltransferase (DLAT) and DLST of the TCA cycle, inducing abnormal oligomerization of lipoylated proteins, triggering proteotoxic stress and subsequent cell death [39]. Given that changes in the lipoylation status of DLST are closely associated with cuproptosis, we further explored the impact of DLST succinylation on cuproptosis sensitivity. After treatment of H1299, A549, and H358 cells with the cuproptosis inducer ES‐Cu, tumor cell growth was inhibited. Since H1299 and H358 cells exhibited lower IC_50_ values (7.27 and 3.745 nM, respectively) and higher drug sensitivity compared to A549 cells (90 nM), these two cell lines were selected for subsequent experiments (Figure 6A). Under ES‐Cu treatment of H1299‐shDLST/WT, H1299‐shDLST/K409R, H358‐shDLST/WT, and H358‐shDLST/K409R cells, CCK‐8 and colony formation assay results showed that DLST K409R significantly reduced cell viability, indicating a marked increase in cuproptosis sensitivity (Figure 6B,C). Then, when the cells were treated with ferrostatin‐1, necrostatin‐1, ZVAD‐FMK, and copper chelators TTM as ferroptosis, necroptosis, apoptosis, and cuproptosis inhibitors, the results showed that only TTM exerted a significant inhibitory effect on copper‐induced cell death (Figure 6D). Further, TEM analysis revealed that in H358‐shDLST/WT cells, the mitochondrial matrix density was moderate with no obvious edema. In contrast, H358‐shDLST/K409R cells displayed a certain degree of mitochondrial swelling and cristae fragmentation. After combined treatment with ES‐Cu, H358‐shDLST/WT cells showed reduced mitochondrial size, suggesting a certain level of mild damage, whereas in H358‐shDLST/K409R cells, mitochondrial shrinkage, membrane thickening, disappearance of cristae structures, and significant expansion of the endoplasmic reticulum were observed (Figure 6E). These findings indicate that under ES‐Cu treatment, desuccinylation of DLST K409 could enhance cellular cuproptosis. Furthermore, Western blot analysis using non‐denaturing gel electrophoresis demonstrated that the desuccinylation promoted DLST lipoylation (Figure 6F). Meanwhile, we observed that after ES‐Cu treatment, the oligomerization level of DLST was increased in the shDLST/K409R group (Figure 6G). These results suggest that DLST K409succ could suppress its lipoylation. In addition, the molecular docking analysis were performed. The results showed that the lipoyl group exhibited strong binding affinity for DLST. Moreover, desuccinylation at DLST K409 enhanced the affinity between DLST and the lipoyl group (Figure S6). Accumulated studies have demonstrated that in the process of protein lipoylation modification is primarily regulated by LIPT1, LIPT2, FDX1, and LIAS, among which LIPT1 and LIAS exert more direct effects [40, 41]. Therefore, we further co‐transfected DLST with LIAS or LIPT1 into 293T cells. Co‐IP assays revealed an interaction between DLST and LIPT1, and desuccinylation at DLST K409 did not affect this binding. Interestingly, wild‐type DLST barely interacted with LIAS, whereas desuccinylation of DLST K409 significantly strengthened their interaction (Figure 6H). Collectively, these results suggest that DLST K409 succinylation inhibits its lipoylation is closely related to the reduction of the affinity between DLST and lipoyl group, and the blockade of the binding between DLST and LIAS.

We further measured mitochondrial copper accumulation via ICP‐MS. The data indicated that copper levels were markedly elevated in LUAD cells expressing the DLST K409R mutant compared with those expressing DLST‐WT (Figure 6I). Xenograft tumor experiments also demonstrated that compared with DLST WT, DLST K409R inhibits tumor growth, and the combination of DLST K409R with ES‐Cu can further suppress tumor growth (Figure 6J–M). During cuproptosis, intracellular protein homeostasis is disrupted, which in turn activates the cellular stress response and induces increased expression of HSP70. Therefore, some studies suggest that HSP70 can be used as a marker for cuproptosis [42]. The IHC staining results showed that compared with the shDLST/WT+ES‐Cu group, the expression of mitochondrial marker TOM20 was decreased, while the expression of HSP70 was increased in shDLST/K409R+ES‐Cu group (Figure 6N). These results suggest that DLST K409succ may promote cuproptosis resistance in LUAD cells by inhibiting its lipoylation modification.

### 3.7 Small‐Molecule Inhibitor SI409‐1 Targeting DLST K409 Effectively Inhibits the Malignant Progression of LUAD

**FIGURE 7:**
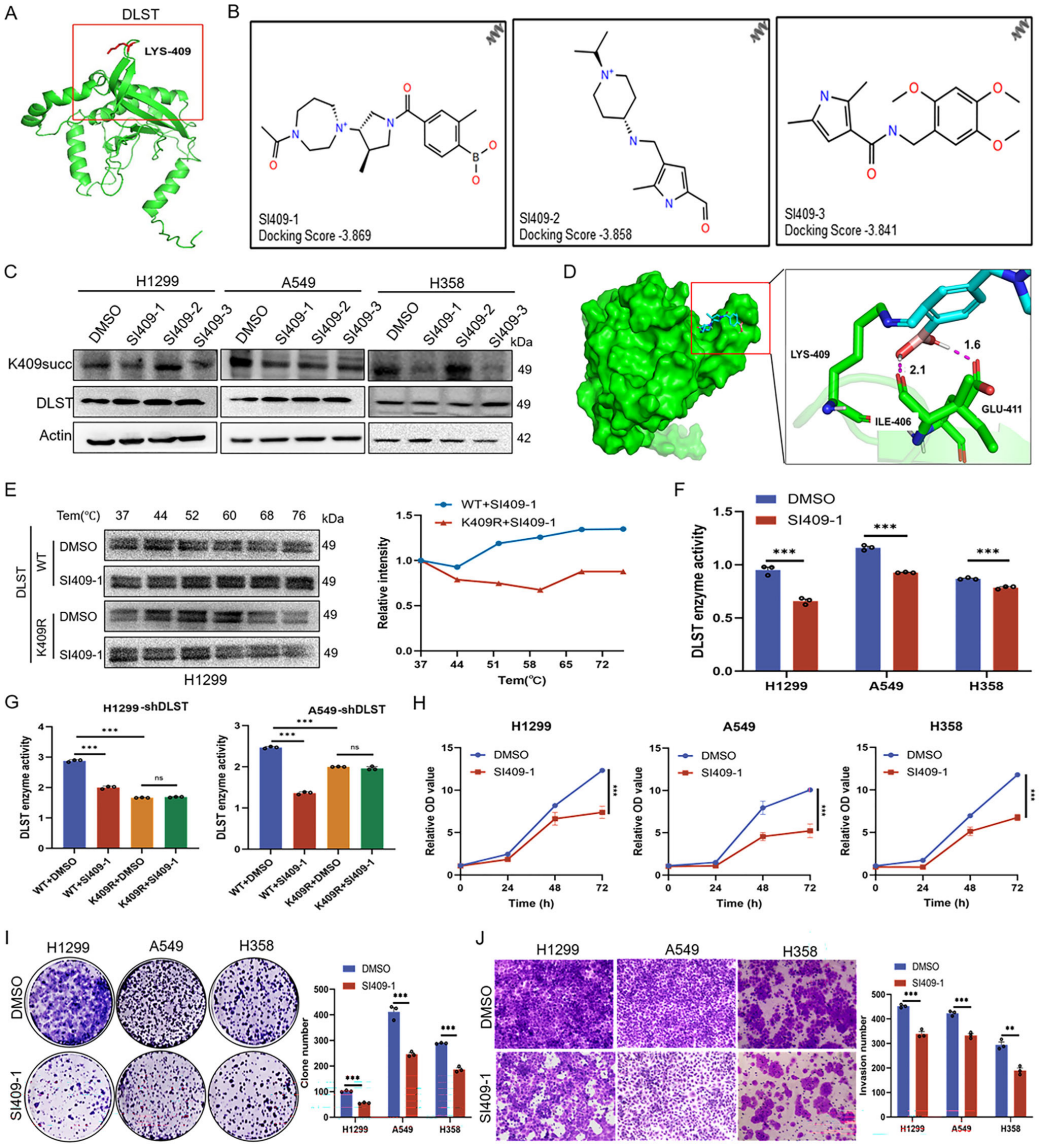
Small‐molecule inhibitor SI409‐1 targeting DLST K409 effectively inhibits the malignant progression of LUAD. (A) Covalent docking site of the DLST protein. (B) Chemical structures and docking scores of the top 3 ranked compounds. (C) Western blot analysis of DLST K409 succinylation in LUAD cells treated with the top three small‐molecule inhibitors. (D) Molecular docking analysis of the interaction between SI409‐1 and DLST K409. (E) CETSA experiment analysis the combination of SI409‐1 with DLST. (F) After treating LUAD cells with SI409‐1 (50 µM) for 24 h, the enzymatic activity of DLST was measured using the α‐KGDHC enzyme activity assay kit (*n* = 3). (G) After treating H1299‐shDLST/WT, H1299‐shDLST/K409R, A549‐shDLST/WT, and A549‐shDLST/K409R cells with DMSO or SI409‐1 (50 µM) for 24 h respectively, the enzymatic activity of DLST was measured using the α‐KGDHC enzyme activity assay kit (*n* = 3). After treating H1299, A549, and H358 cells with SI409‐1 (50 µM) for indicated time, the cell proliferation was detected by the CCK‐8 (H) and colony formation assay (I), and the cell invasion (J) was detected by transwell assay (*n* = 3). Data were shown as the mean ± SD of at least three independent experiments. **p < 0.01, ***p < 0.001. Statistical significance was determined by two‐tailed unpaired Student's *t*‐test (F, H) and the two‐way ANOVA (G).

Given the important role of DLST K409succ in the malignant progression of LUAD, the development of a small‐molecule inhibitor targeting DLST K409 will provide a novel intervention approach for LUAD. First, we conducted a high‐throughput virtual screening based on molecular structure (Figure S7A). Human DLST structure was downloaded from RCSB PDB, and its K409‐centered covalent binding pocket was analyzed (Figure 7A). The HY‐L0076V library (10,480 small molecules) was processed into 3D structures using Schrödinger's LigPrep Module, top 100 compounds were selected via Covalent Docking (Lys 409 as target, Michael Addition, virtual screening mode). The top 3 potential inhibitors were synthesized (Figure 7B). We evaluated the activity of these three inhibitors at multiple concentrations, and the results showed that all three inhibitors exerted a certain inhibitory effect on tumor cell viability (Figure S7B). Further Western blot results showed that, compared with the other two small‐molecule inhibitors, SI409‐1 significantly inhibited DLST K409succ in LUAD cells without altering the global cellular succinylation level (Figure 7C, Figure S7C). SI409‐1 and DLST K409 binding pockets have strong binding affinity (Figure 7D). To confirm whether SI409‐1 directly binds to DLST, we employed the CETSA assay to simulate intracellular binding between small molecules and proteins [43]. The results demonstrated that under various temperature conditions, the stability of DLST in samples pre‐treated with SI409‐1 was significantly higher than that in the DMSO control group, confirming direct binding between SI409‐1 and DLST. More importantly, this binding was markedly reduced in the DLST K409R mutant protein group (Figure 7E).

We then evaluated the effects of SI409‐1 on enzyme activity and found that it significantly inhibits DLST enzymatic activity in LUAD cells (Figure 7F). In DLST‐knockdown A549 and H1299 cells, we overexpressed DLST‐WT and the K409R mutant, respectively. The cells were then treated with DMSO or SI409‐1 and α‐KGDHC enzymatic activity was measured. The results showed that SI409‐1 reduced the enzyme activity in the DLST‐WT group compared with DMSO treatment. However, this inhibitory effect was abolished in cells expressing the DLST K409R mutant (Figure 7G). Consistent with expectations, SI409‐1 displayed obvious suppressive effects on all three LUAD cell types, markedly reducing their proliferation and invasion (Figure 7H–J). These results indicate that SI409‐1 can inhibit the malignant progression of LUAD cells by suppressing DLST K409succ.

**FIGURE 8:**
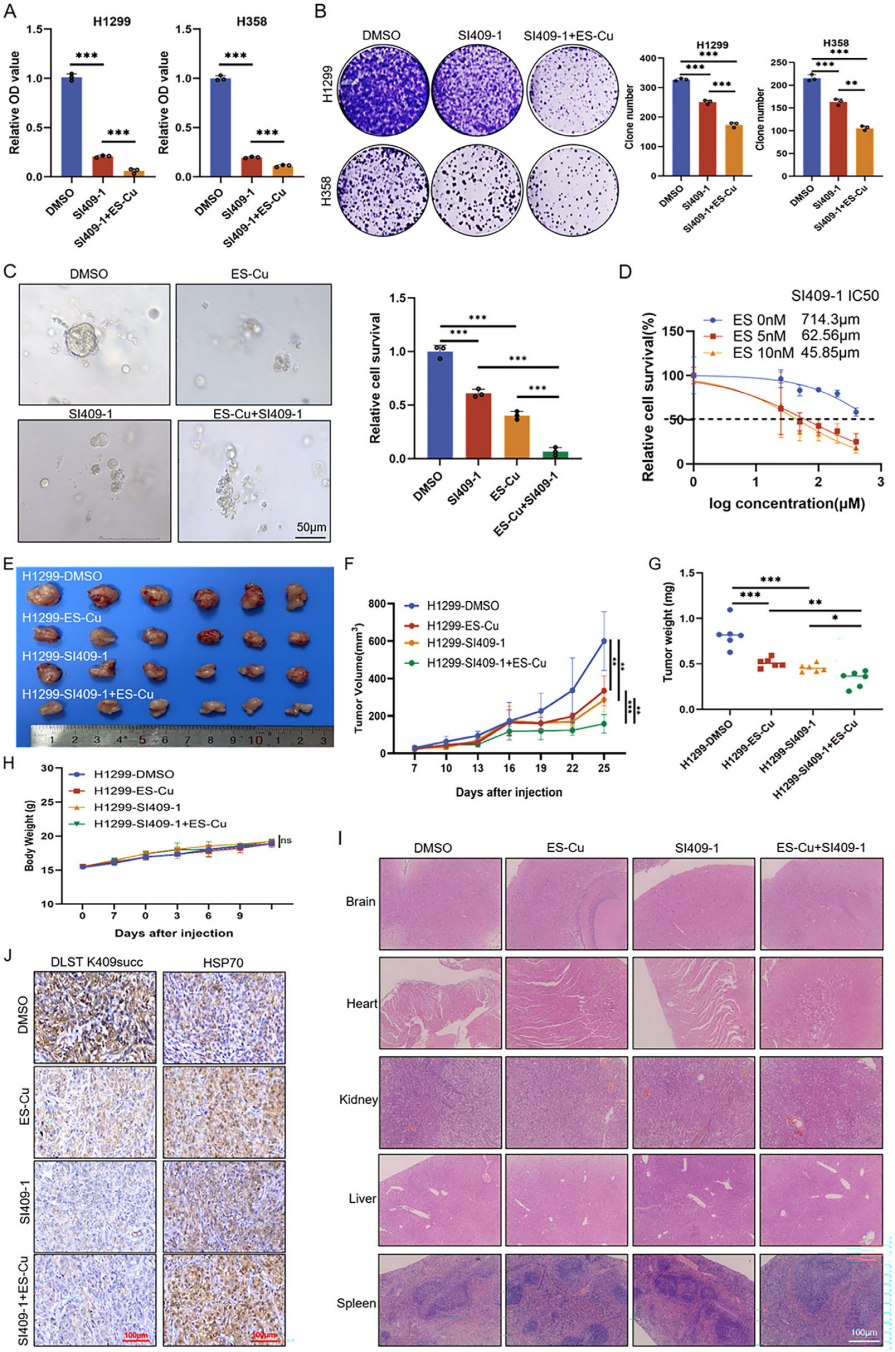
SI409‐1 enhances the cuproptosis sensitivity of LUAD. Treat LUAD cells with SI409‐1 (50 µM) in combination with ES‐Cu (5 nM ES+1 µM CuCl_2_), (A) CCK‐8 and (B) Colony formation assay to detect the proliferation of LUAD cells. (C) LUAD PDOs were treated with different concentrations of SI409‐1 and ES‐Cu for 5 days, and PDO viability was assessed using the CCK‐8 assay. Representative images of PDOs under different treatment conditions are shown. (D) IC_50_ values of SI409‐1 were determined across all experimental conditions in LUAD PDOs. After 5×10^6^ H1299 cells were injected subcutaneously into 5‐week‐old nude mice and randomly divided into DMSO, ES‐Cu (ES:5 mg/kg + CuCl_2_:0.06 mg/kg), SI409‐1 (15 mg/kg), and ES‐Cu+SI409‐1 group (*n* = 6), the tumor growth (E), tumor volume (F), and tumor weight (G) were monitored. (H) Changes in mouse body weight during the administration period. (I) Representative images of normal organs in each group of mice stained with H&E. (J) Representative images of IHC staining of DLST K409succ and HSP70 in xenograft tumor tissues. Data were shown as the mean ± SD of at least three independent experiments. *p < 0.05, **p < 0.01, ***p < 0.001. Statistical significance was determined by two‐tailed unpaired Student's *t*‐test (B, G) and the two‐way ANOVA (A, F, H).

We further investigated the combined effect of SI409‐1 and ES‐Cu on LUAD cells. The results of the CCK‐8 and colony formation assay showed that the combined treatment with SI409‐1 and ES‐Cu significantly inhibited the proliferation of LUAD cells (Figure 8A,B). We successfully established patient‐derived organoid (PDO) models using surgically resected clinical LUAD tissues. To investigate the responsiveness of PDOs to SI409‐1 and combination therapy, we performed dose‐gradient drug screening. The data demonstrated that both SI409‐1 and ES‐Cu could significantly inhibit the proliferation of PDOs. Meanwhile, SI409‐1 could sensitize PDOs to ES‐Cu treatment (Figure 8C,D). In xenografts, the individual treatment groups of SI409‐1 or ES‐Cu could inhibit the growth of subcutaneous xenografts in mice, while the combined treatment group could further reduce tumor growth (Figure 8E–G). Meanwhile, there was no significant change in the weight of the mice, indicating that these treatments did not have significant toxic side effects (Figure 8H). To further evaluate potential systemic toxicity, major organs were harvested for histological analysis via H&E staining. No obvious pathological lesions were observed in the brain, liver, kidney, heart, spleen and other vital organs of treated mice, suggesting that the tested agents possess favorable safety with low systemic toxicity in vivo (Figure 8I). The IHC staining results showed that SI409‐1 could effectively reduce DLST K409succ, and increase the expression level of HSP70 after combined treatment of SI409 and ES‐Cu (Figure 8J). These results indicate that SI409‐1 could enhance the sensitivity of LUAD to cuproptosis and significantly inhibit the malignant progression of LUAD.

## 4 Discussion

The TCA cycle, serving as a pivotal hub for energy production and material transformation, demonstrates that dysregulation of its key enzymes is closely associated with tumor development. As a crucial form of post‐translational protein modification, succinylation dynamically regulates the activity and localization of metabolic enzymes, playing a critical role in cellular metabolic remodeling and tumorigenesis [44]. In the present study, we found that DLST, the rate‐limiting enzyme of the TCA cycle, exhibited higher succinylation levels in LUAD compared to adjacent normal tissues. CPT1A promoted succinylation at the DLST K409 site, which facilitated the malignant progression of LUAD by enhancing its enzymatic activity and mitochondrial metabolic function.

The regulation of succinylation is primarily categorized into two mechanisms: enzymatic and non‐enzymatic reactions. Early studies predominantly attributed this process to non‐enzymatic reactions mediated by succinyl‐CoA [45]. However, subsequent studies have revealed a regulatory network involving “writers” (succinyltransferases) and “erasers” (desuccinylases), and the enzymatic reactions play more important role in the regulation of succinylation [45]. CPT1A, HAT1, and KAT2A have been confirmed to possess succinylation transferase activity, enabling them to catalyze succinylation in both histone and non‐histone proteins [26, 32, 46]. CPT1A is an enzyme localized in the outer mitochondrial membrane. Its classical function is to catalyze the combination of long‐chain fatty acids with carnitine to form acylcarnitines, serving as the rate‐limiting enzyme in the fatty acid β‐oxidation pathway and the primary regulatory point governing the entry of fatty acids into mitochondria for catabolism [47]. In 2018, Kurmi et al. first identified and demonstrated that CPT1A possesses succinyltransferase activity by employing an amino acid‐based proteomics approach with stable isotope labeling in cell culture [32]. Chen et al. reported that in melanoma, CPT1A can catalyze the succinylation modification of PD‐L1 at position K129, inducing its degradation and thereby relieving the inhibitory effect of PD‐L1 on CD8+ T cells, providing a novel target for reversing immune therapy resistance [48]. In this study, combined with direct enzyme activity assays, kinetic analysis and succinylation analysis, we demonstrated that CPT1A mediates DLST K409 succinylation via a non‐canonical mechanism. Furthermore, we elucidated that CPT1A‐regulated succinylation of DLST cross‐talks with its lipoylation, thereby modulating the sensitivity of LUAD cells to cuproptosis. Furthermore, since succinyl‐CoA can mediate the succinylation of other molecules and DLST is also regarded as a succinyltransferase in some studies [49, 50], the positive feedback regarding DLST succinylation thus requires further investigation in our subsequent research.

Mitochondria are the core organelles for energy metabolism in eukaryotic cells, and also serve as hubs mediating cell death processes and signal transduction [51]. Cuproptosis, a newly identified form of copper‐dependent regulated cell death, primarily occurs in mitochondria. Studies have shown that its mechanism mainly involves excessive copper ions binding to lipoylated proteins in the TCA cycle—such as DLAT or DLST. This binding induces abnormal oligomerization of lipoylated proteins and reduced stability of iron‐sulfur cluster proteins, triggering proteotoxic stress and ultimately leading to cell death [39]. Recently, cuproptosis has garnered significant attention in tumor research due to its potential as a key therapeutic strategy [52, 53, 54]. For example, the lactylation of METTL16 at K229 upregulates FDX1 protein expression via m6A modification on FDX1 mRNA and serves as a pathway for triggering cuproptosis under copper stress. Combining the ES‐Cu with AGK2, which can enhance the METTL16 lactylation, significantly induces cuproptosis in gastric tumors in vitro and in vivo [55]. Enzalutamide promotes the aggregation of lipoylated proteins and the instability of iron‐sulfur cluster proteins. The combined use of enzalutamide and ES‐Cu can synergistically induce cuproptosis in castration‐resistant prostate cancer cells [56]. Recent studies have found that ferroptosis inducers sorafenib and erastin can upregulate DLAT protein lipoylation, thereby promoting cuproptosis in primary liver cancer cells, suggesting a new therapeutic strategy combining ferroptosis inducers and copper ion carriers [57]. In our study, we clarified that DLST is a molecule mediating cuproptosis, the succinylation of DLST inhibits cuproptosis via delipidation, while DLST K409R may enhance the sensitivity of LUAD cells to cuproptosis by increasing its protein lipoylation, thereby providing a target for novel interventions. However, the current functional and mechanistic experiments were mainly performed using plasmids carrying the DLST K409 succinylation mimetic mutant. Given the differences in modification regulation between exogenous mutant plasmids and endogenous cellular proteins, we cannot fully clarify the dynamic regulation of DLST succinylation and its cross‐talk with other protein modifications in tumor cells, which requires further investigation.

Since α‐KGDHC exerts an oncogenic effect, inhibitors targeting α‐KGDHC have been proven to exhibit favorable anti‐tumor activity. Devimistat, a lipoic acid analog that inhibits the α‐KGDHC, showed in a phase I clinical trial in relapsed/refractory acute myeloid leukemia (AML) that AML patients receiving Devimistat combined with chemotherapy achieved an overall response rate (ORR) of 50% and a median OS of 6.7 month [58]. Sandra Atlante et al. found that the α‐KGDHC inhibitor AA6 could activate TET enzymes to promote DNA demethylation, upregulate the miR‐200 family, suppress EMT transcription factors, and thereby reduce the expression of MMP3, ultimately inhibiting lung metastasis of breast cancer [59]. Given the important role of DLST in the α‐KGDHC and the regulatory role of DLST K409succ in its enzymatic activity and cuproptosis resistance, in this study, we identified small‐molecule inhibitor SI409‐1 targeting DLST K409 via virtual screening and demonstrated its ability to inhibit DLST K409succ. In vitro and in vivo experiments have found that SI409‐1 could inhibit the LUAD malignant progression and enhance the cuproptosis sensitivity. This provides new predictive targets and intervention approaches for the treatment of LUAD. However, research on the small molecule inhibitor SI409‐1 remains in its preliminary exploratory phase. This study preliminarily verified the anti‐tumor efficacy of SI409‐1 in vivo. Notably, we did not conduct systematic pharmacokinetic analyses, including plasma half‐life, bioavailability, and tissue distribution, nor did we explore the correlation between pharmacokinetics and pharmacodynamics. Such data are essential for evaluating the drugability of SI409‐1 and will be prioritized in future preclinical investigations.

Mitochondria serve not only as the core organelles for metabolism and energy conversion, but also as key platforms mediating cellular death such as apoptosis, autophagy, and cuproptosis. In recent years, research on tumor metabolism has expanded beyond regulating intrinsic pathways in cancer cells to explore how tumor metabolism influences other tumor characteristics, including immune regulation. Accumulating evidence indicates that mitochondrial metabolic reprogramming promotes tumor progression by triggering metabolite‐dependent immunosuppression in the tumor microenvironment (TME) [60, 61]. The TCA cycle metabolite α‐KG, for instance, upregulates fatty acid oxidation via epigenetic regulation and induces differentiation of immunosuppressive M2 macrophages [62]. Meanwhile, it is currently believed that cuproptosis plays a crucial regulatory role in the TME with immune cell infiltration [63]. In lung cancer, cuproptosis is pivotal for shaping tumor immune microenvironment (TIME) through the gene regulation of immune checkpoints as well as the modulation of tumor infiltrating leukocytes [64]. Our present work reveals that CPT1A‐regulated DLST K409 succinylation promotes tumor progression by remodeling mitochondrial metabolism and regulating cuproptosis sensitivity in LUAD cells. Future studies will utilize animal models to explore the impact of DLST K409 succinylation on TIME under immune‐competent conditions, and analyze the effects of SI409‐1 combined with immunotherapy, such as anti‐PD‐1 therapy.

**FIGURE 9:**
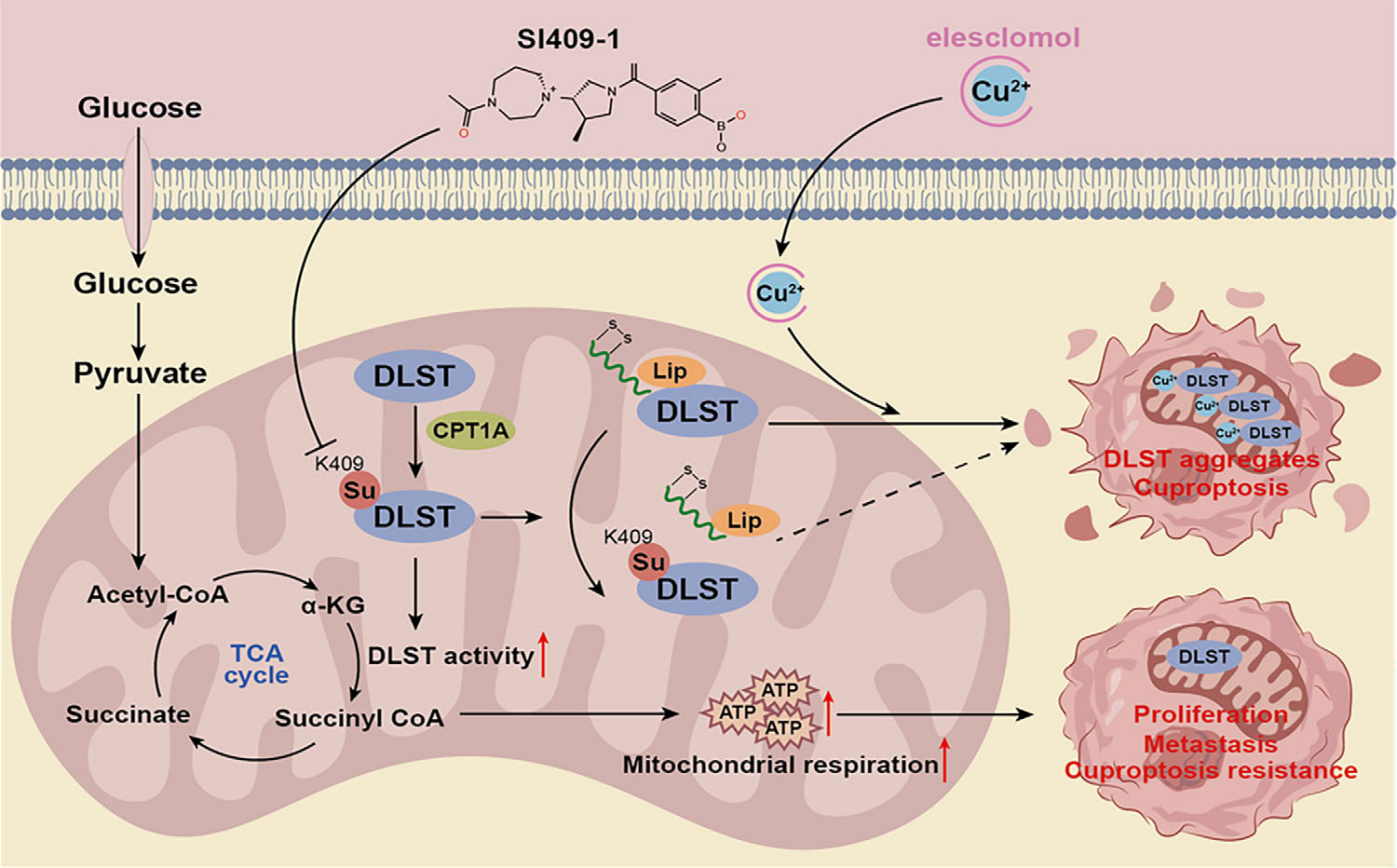
Schematic diagram of the mechanism by which DLST K409succ promotes malignant progression of LUAD by remodeling mitochondrial metabolism and cuproptosis resistance.

In conclusion, we demonstrate for the first time the mechanism by which CPT1A regulates the succinylation of the TCA cycle rate‐limiting enzyme DLST to promote LUAD malignant progression. Specifically, CPT1A‐mediated DLST K409succ enhances mitochondrial metabolism, proliferation, and invasion of LUAD cells by increasing DLST enzymatic activity. Meanwhile, K409succ also promotes cuproptosis resistance by reducing DLST lipoylation. The small‐molecule inhibitor SI409‐1, which targets DLST K409succ, interferes with tumor malignant progression by reversing this process and potentiating the effects of cuproptosis inducers (Figure 9).

## Author Contributions

L.Y., Q.Z., and X.L. conceived and designed the study. X.L., P.Z., X.P., Q.L., and W.Z. performed experiment. X.L., P.Z., X.P., R.W., L.S., and J.P. analyzed data. X.L. and L.Y. wrote the manuscript. All authors read and approved the final manuscript.

## Funding

This work was supported by grants from the National Natural Science Foundation of China (82573929, 82372683), the Natural Science Foundation of Hunan Province (2025JJ60588, 2026JJ70063), China Postdoctoral Science Foundation (2025M781427), and the Fundamental Research Funds for the Central Universities of Central South University (1053320241663).

## Conflicts of Interest

The authors declare no conflicts of interest.

## Ethics Statement

The study was approved by the Medical Ethics Committee of Xiangya Hospital, Central South University and all samples were collected with the patients’ informed consent. All animal studies were approved by the Institutional Animal Care and Use Committee of Central South University.

## Supporting information




**Supporting File**: advs77528‐sup‐0001‐SuppMat.docx.

## Data Availability

The mass spectrometry proteomics and succinylome data of this study to the ProteomeXchange Consortium (https://proteomecentral.proteomexchange.org) via the iProX partner repository with the dataset identifier PXD079698. Other data supporting the findings of this study are available within the paper and its supplementary materials. Additional information is available from the corresponding author upon reasonable request.
