# Supplementary material for: DLST Succinylation‐Mediated Mitochondrial Metabolic Remodeling and Cuproptosis Resistance Promote Malignant Progression of Lung Adenocarcinoma
Source: Adv Sci (Weinh). 2026 Aug 30:e77528. Online ahead of print. doi: 10.1002/advs.77528 (PMC13526706; doi:10.1002/advs.77528)
Supplement: Supplementary file 1 [file ADVS-9999-e77528-s001.docx]

**Supplementary Materials**

**DLST** **succinylation-mediated mitochondrial metabolic remodelling and cuproptosis** **resistance promote malignant progression of lung adenocarcinoma**

Xuanxuan Li, Peijun Zhou, Xingzhi Peng, Qixin Liu, Wenhui Zeng, Rui Wang, Liangfang Shen, Jinwu Peng, Qin Zhou, Lifang Yang

**Content**

● **Figure. S1.** Upregulation of succinylation modification in LUAD tissue samples.

● **Figure. S2.** Generation of site-specific plasmids and antibody against DLST K409succ.

● **Figure. S3.** Knockdown of DLST inhibits the malignant progression of LUAD cells.

● **Figure. S4.** Succinylation of DLST affects mitochondrial metabolism in LUAD cells.

● **Figure. S5.** Knockdown of CPT1A inhibits the malignant progression of LUAD cells.

● **Figure. S6.** The DLST K409R mutation impedes the binding of lipoyl-AMP to DLST.

● **Figure. S7.** Small-molecule inhibitor targeting the DLST K409.

● **Table S1.** Clinical characteristics of LUAD patients for succinylation proteomics analysis.

● **Table S2.** Clinical characteristics of LUAD patients for IHC staining analysis.

● **Table S3.** Primers for construction of expression plasmids.

● **Table S4.** The target sequences of shRNAs.

**● Table S5** Clinicopathological information of the LUAD PDO patient.

**Supplementary Figures**

**Figure S1** **Upregulation of succinylation modification in LUAD tissue samples.** (A) Pearson correlation analysis among different samples by 4D label-free lysine succinylation proteomics mass spectrometry analysis. (B) Principal Component Analysis (PCA) plots of lysine succinylation proteomics data. (C) Volcano plot of differentially succinylated proteins. (D) Subcellular localization of succinylated proteins. (E) Analysis of DLST expression in LUAD using the CPTAC proteomic database. (F) Kaplan-Meier curve showing the survival curve of LUAD patients with DLST expression using TCGA-LUAD database.

**Figure S2** **Generation of site-specific plasmids and antibody against DLST K409succ.** (A) Identification of DLST succinylation sites in LUAD tissues via lysine succinylation proteomics mass spectrometry analysis. (B) Sanger DNA sequencing identified the mutation sites of DLST-WT, DLST-K409R, and DLST-K409E. (C) Verification of DLST K409succ in HeLa and HepG2 cells using antibodies Ab1, Ab2, and Ab3, respectively. (D) Identification of the DLST K409succ antibody Ab-3 via dot blot assay. (E) Identification of the DLST K409succ antibody Ab-3 via ELISA.

**Figure S3** **Knockdown of DLST inhibits the malignant progression of LUAD cells.** (A) Verification of knockdown efficiency of shDLST#1 and shDLST#2 in H1299 and A549 cells. (B) CCK-8 and (C) Colony formation assay to detect cell proliferation after DLST knockdown (n=3). (D) Wound-healing assay to detect cell migration after DLST knockdown (n=3). (E) Transwell assay to detect cell invasion after DLST knockdown (n=3). Data were shown as the mean ± SD of at least three independent experiments. NC, negative control, *p < 0.05, **p < 0.01, ***p < 0.001. Statistical significance was determined by two-tailed unpaired Student’s t-test (C,D,E) and the two-way ANOVA (B).

**Figure S4** **Succinylation of DLST affects mitochondrial metabolism in LUAD cells.** (A) PCA plot of non-targeted metabolomics data. (B) Differential metabolites between A549-shDLST/WT and A549-shDLST/K409R groups. (C) Glycolysis was detected using the ECAR assay in the stable cell lines H1299-shDLST/WT, H1299-shDLST/K409R, H1299-shDLST/K409E, A549-shDLST/WT, A549-shDLST/K409R, and A549-shDLST/K409E (n=3). (D) ATP production rate in glycolysis (n=3). H1299-shDLST/WT, H1299-shDLST/K409R, A549-shDLST/WT and A549-shDLST/K409R cells were treated with 2 mM dimethyl succinate. Cell proliferation was evaluated by CCK-8 assay (n=4) (E) and colony formation assay (n=3) (F). Transwell assay was performed to detect the invasive ability of LUAD cells (n=3) (G).  Data were shown as the mean ± SD of at least three independent experiments. ns, no significant difference. ***p* < 0.01, ****p* < 0.001.

**Figure S5** **Knockdown of CPT1A inhibits the malignant progression of LUAD cells.** (A) Analysis of CPT1A protein expression in LUAD based on the CPTAC database. (B) Kaplan-Meier survival analysis was performed based on CPT1A gene expression levels. (C) Western Blot to detect CPT1A expression in BEAS-2B, H1299, A549, H358 and PC9 cells. (D) CCK-8 and (E) colony formation assay to detect cell proliferation after CPT1A knockdown (n=3). (F) Wound-healing assay to detect cell migration after CPT1A knockdown (n=3). (G) Transwell assay to detect cell invasion after CPT1A knockdown (n=3). Data were shown as the mean ± SD of at least three independent experiments. (H) The correlation between CPT1A expression and sensitivity to commonly used drugs in LUAD was analyzed using the GDSC database. (I) IHC scores of DLST K409succ in 70 clinical samples stratified by EGFR mutation status. NC, negative control, **p* < 0.05, ***p* < 0.01, ****p* < 0.001. Statistical significance was determined by two-tailed unpaired Student’s t-test (B,C,D) and the two-way ANOVA (D).

**Figure. S6.** The DLST K409R mutation impedes the binding of lipoyl-AMP to DLST. Molecular docking of lipoyl-AMP with DLST-WT and DLST-K409R was performed using CB-Dock2.

**Figure S7** **Small-molecule inhibitor targeting the DLST K409.** (A) Virtual screening workflow based on molecular docking. (B)IC₅₀ of small-molecule inhibitor targeting the DLST K409 site in LUAD cells. (C) After 24-hour treatment with DMSO or SI409-1 in H1299 and A549 cells, pan-succinyl lysine antibody was used to examine protein succinylation.

**Supplementary Tables**

**Table S1** **Clinical characteristics of LUAD patients for succinylation proteomics analysis.**

| Case | Gender | Age | TNM stage | Clinical stage |
| --- | --- | --- | --- | --- |
| Case1 | Male | 71 | T1bN0M0 | IA |
| Case2 | Male | 61 | T1aN0M0 | IA |
| Case3 | Female | 51 | T1bN0M0 | IA |
| Case4 | Male | 57 | T1bN1M0 | IIA |
| Case5 | Male | 69 | T1cN0M0 | IA |

**Table S2** **Clinical characteristics of LUAD patients for IHC staining analysis.**

| Characteristics | Number of patients (%) | |
| --- | --- | --- |
| Overall | | 70 |
| Gender | |  |
| Male | | 25（35.71%） |
| Female | | 45（64.29%） |
| Age | |  |
| ≥45 | | 67（95.71%） |
| ＜45 | | 3（4.28%） |
| Smoking | |  |
| Yes | | 25（35.71%） |
| No | | 45（64.29%） |
| Tumor size | |  |
| ＜2 | | 36（51.43%） |
| ≥2 | | 34（48.57%） |
| T | |  |
| T1 | | 31（44.29%） |
| T2 | | 34（48.57%） |
| T3 | | 2（2.86%） |
| T4 | | 3（4.29%） |
| N | |  |
| N0 | | 61（87.14%） |
| N1orN2 | | 9（12.86%） |
| M | |  |
| M0 | | 69（98.57%） |
| M1 | | 1（1.43%） |
| Stage | |  |
| I | | 57（81.42%） |
| II | | 3（4.29%） |
| III | | 9（12.86%） |
| IV | | 1（1.43%） |
| EGFR | |  |
| mutations | | 18（25.71%） |
| non-mutations | | 52（74.29%） |

**Table S3** **Primers for construction of expression plasmids.**

| ID | Sequence (5’ - 3’) |
| --- | --- |
| pcDNA3.1-DLST-F | tgctggatatctgcagaattcATGCTGTCCCGATCCCGC |
| pcDNA3.1-DLST-R | cttggtaccgagctcggatccAAGATCCAGGAGGAGGACTCTGG |
| pCDH-3×Flag-DLST-F | gattctagagctagcgaattcATGCTGTCCCGATCCCGC |
| pCDH-3×Flag-DLST-R | atggtctttgtagtcggatccAAGATCCAGGAGGAGGACTCTGG |
| pCDH-3×Flag-DLST-K409R-F | ATAGGAGGCAGGGTAGAGGTGCGGCCCATGAT |
| pCDH-3×Flag-DLST-K409R-R | TCTACCCTGCCTCCTATAGCCACTGGCCTGTC |
| pCDH-3×Flag -DLST-K409E-F | ATAGGAGGCGAGGTAGAGGTGCGGCCCATGAT |
| pCDH-3×Flag-DLST-K409E-R | TCTACCTCGCCTCCTATAGCCACTGGCCTGTC |
| GST-DLST-F | ccgcgtggatccccggaattcATGCTGTCCCGATCCCGC |
| GST-DLST-R | ctcgagtcgacccgggaattcAAGATCCAGGAGGAGGACTCTGG |
| His-CPT1A-H473A-F | AACGCTGAAgccTCCTGGGCAGATGCGCCGAT |
| His-CPT1A-H473A-R | CAGGAggcTTCAGCGTTGAGGCCCATCTTCCC |

**Table S4** **The target sequences of shRNAs.**

| ID | Sequence (5’ - 3’) |
| --- | --- |
| shNC | TTCTCCGAACGTGTCACGT |
| shDLST#1 | CGAAAGAATGAACTTGCCATT |
| shDLST#2 | CGCTCTTTGGAACACCCATTA |
| shCPT1A | GCCATGAAGCTCTTAGACAAA |

**Table S5 Clinicopathological information of the LUAD PDO patient.**

| Case | Gender | Age | TNM stage | Clinical stage |
| --- | --- | --- | --- | --- |
| Case1 | Male | 58 | T2bN0M0 | IIA |
